# Proton FLASH Exposure Preserves Gut Commensal Microbiomes and Spares Intestinal Stem Cells

**DOI:** 10.1002/advs.202519249

**Published:** 2026-03-28

**Authors:** Rishi Man Chugh, Payel Bhanja, Emily Schueddig, Jufri Setianegara, Yuting Lin, Kenny Guida, Shujah Rehman, Stacey Krepel, Devin Koestler, Ronald C. Chen, Katherine L. Cook, Subhrajit Saha

**Affiliations:** ^1^ Department of Radiation Oncology University of Kansas Medical Center Kansas City Kansas USA; ^2^ Department of Biostatistics & Data Science University of Kansas Medical Center Kansas City Kansas USA; ^3^ Department of Cancer Biology and Surgery Wake Forest University School of Medicine Winston‐Salem North Carolina USA; ^4^ Department of Cancer Biology University of Kansas Medical Center Kansas City Kansas USA; ^5^ Department of Pathology and Laboratory Medicine University of Kansas Medical Center kansas City Kansas USA

**Keywords:** fecal microbiota transplant, gut microbiome, intestinal stem cells (ISCs), proton FLASH/proton CONV irradiation, radiation‐induced intestinal injury, revival stem cells (revSCs), single cell RNA sequencing (scRNA Seq)

## Abstract

Emerging evidence shows that Proton FLASH radiotherapy can spare normal tissues while maintaining anti‐tumor efficacy. However, its impact on intestinal stem cells (ISCs) and the gut microbiome remains unclear. Gut microbiome influences ISC's radiosensitivity. In a mouse model of abdominal irradiation, Proton FLASH exposure exhibited improved survival and less crypt‐villus damage compared to Proton Conventional dose rate. Using scRNA‐sequencing, we demonstrated that Proton FLASH exposure using pulsed pencil beam scanning spares two distinct ISC populations, *Lgr5+* Crypt‐based columnar cells (CBCs) and a *Ly6a+*, *Clu+, Areg+, Anxa2+* revival stem cell (revSC) population—by modulating oxidative stress and cell cycle progression. Analysis of α and β‐diversity demonstrated that Proton FLASH modulates gut microbiota composition without compromising overall species richness. Notably, Proton FLASH‐irradiated mice had higher abundances of *Alistipes sp*. and *Akkermensia sp*., both known for protective effects on ISCs and the intestinal mucosa. The role of microbiome in Proton FLASH‐mediated sparing effect was further confirmed by fecal microbiota transplantation, where Proton FLASH‐donor microbiota demonstrated reduced lethality with protection of crypt villus morphology in recipient mice exposed to Proton Conventional dose rate. Our findings highlight the crucial role of the microbiome in the Proton FLASH‐mediated sparing of the mucosal epithelium.

## Graphical abstract

This study highlights the role of Proton FLASH abdominal irradiation in sparing of intestinal stem cells and preservation of key gut microbial population resulting minimization of radiation toxicity in intestinal epithelium in mice. Our findings support the potential of Proton FLASH to improve the therapeutic ratio for abdominal radiation exposure.

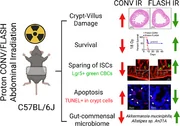

## 1 Introduction

Acute gastrointestinal (GI) toxicity significantly limits the effectiveness of abdominal radiotherapy [1]. Patients with abdominal malignancies often experience severe (grade 3) mucosal toxicity, potentially leading to insufficient tumoricidal radiation doses, early treatment withdrawal, tumor progression or recurrence, and ultimately, a poorer prognosis [2]. The rapid self‐renewal rate of the intestinal epithelium makes it highly susceptible to radiation‐induced DNA damage, resulting in significant radiosensitivity compared to more slowly self‐renewing tissues [3]. Our previous findings suggest that intestinal stem cells, the foundation of epithelial architecture, are key determinants of intestinal epithelium survival following radiation exposure [3, 4].

Radiation dose rate significantly impacts tissue radiosensitivity [5]. Ultra‐high dose rate (FLASH) radiotherapy (RT) (≥40–60 Gy/s) can potentially spare normal tissues while maintaining the anti‐tumor effect, though this effect can be tissue‐dependent [6, 7]. However, the precise biological mechanisms underlying the FLASH effect remain largely unclear. Some studies propose that differential oxygen depletion at ultra‐high dose rates contributes to the selective sparing of healthy tissues in FLASH RT [8, 9, 10]. Specifically, the rapid depletion of oxygen during FLASH RT may minimize the toxic biological effects of free radicals compared to conventional X‐ray radiation by increasing the likelihood of radical–radical recombination due to a transient, high concentration of radicals during a short timeframe [9]. Here, we investigated the biological effects of proton FLASH delivered with the pulsed pencil beam scanning IBA ProteusONE system, reported for the first time in this study.

Intestinal stem cells (ISCs) hierarchy plays a significant role in intestinal epithelial homeostasis and repair [11, 12]. Previous reports suggested that FLASH RT preserves different ISC lineages [7, 13]. However, the mechanism of the FLASH effect on ISCs is not clear. Moreover, FLASH effect on ISC regenerative response, cell cycle, and oxidative stress has not been studied.

Intestinal microbiomes play a significant role in gut health [14]. Radiation‐induced oxidative stress and ROS production in the intestinal epithelium can create an unfavorable environment for the survival of anaerobic commensal bacteria, exerting a selection pressure that reduces their populations and promotes dysbiosis, thereby disrupting the delicate balance of the gut microbiome [15]. Gut microbial dysbiosis substantially contributes to mucosal injury by impairing the regenerative function of ISCs, inducing inflammation, and disrupting the mucosal barrier. Therefore, minimizing mucosal dysbiosis and maintaining a healthy commensal microbiome are crucial for mitigating radiation‐induced intestinal injury [16].

However, the effect of radiation dose rate on mucosal dysbiosis has not been well‐examined. Given that commensal microbiomes are primarily anaerobic, the radiation‐induced production of oxidative radicals may influence the commensal microbiome. Considering the role of FLASH radiotherapy in regulating free radical production, the effect of Proton FLASH on the gut microbiome needs to be investigated in a murine model of abdominal irradiation.

This study demonstrates that Proton FLASH irradiation preserves the gut commensal microbiome with the enrichment of mucoprotective *species*, spares intestinal stem cell lineages, and reduces overall lethality compared to Proton Conventional (CONV) dose rate irradiation.

## 2 Results

### 2.1 Proton FLASH Reduces Severe Morbidity Compared With Proton CONV Dose Rate

**FIGURE 1:**
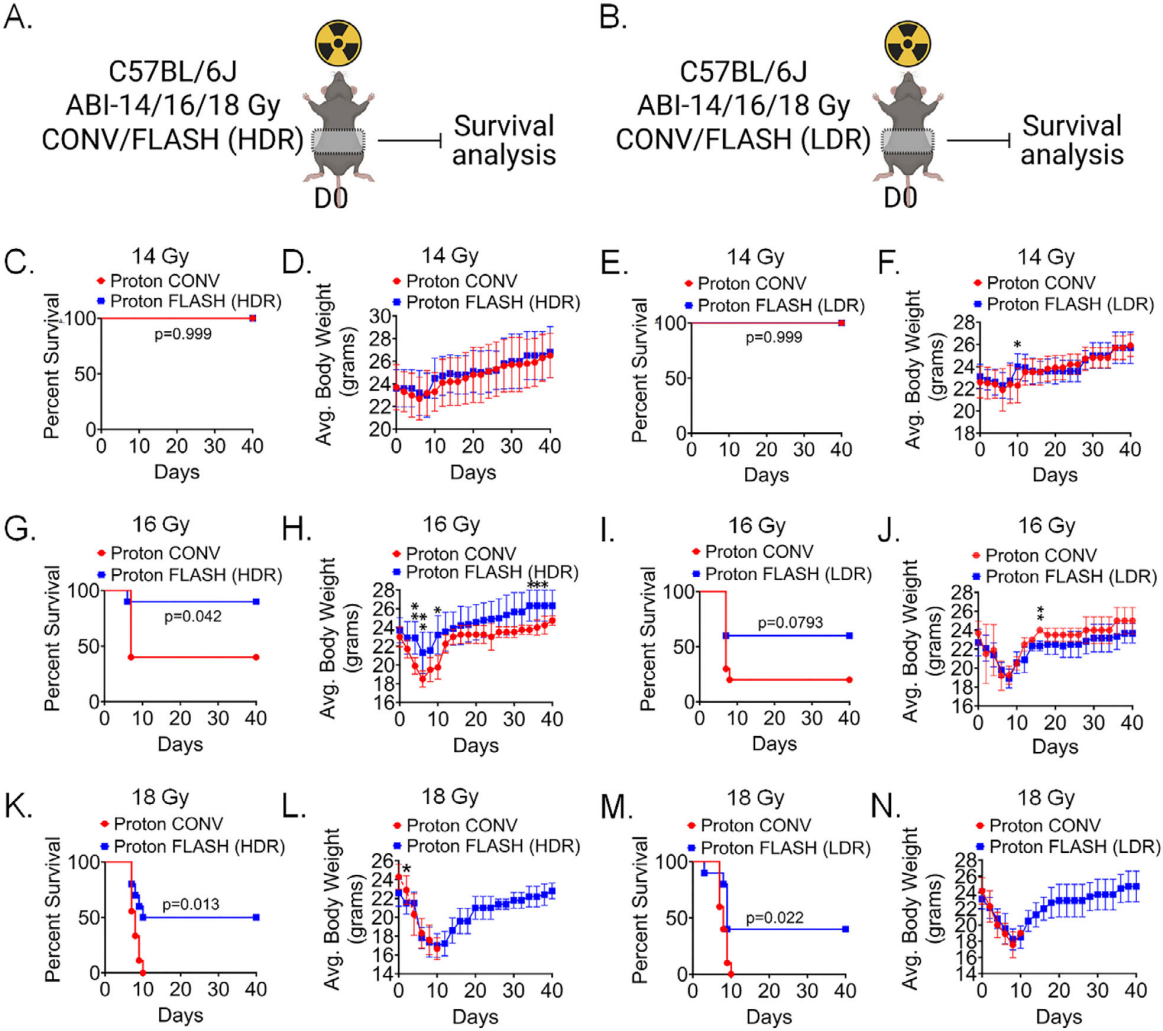
Proton FLASH irradiation reduces morbidity compared with Proton CONV dose rate. A,B) Schematic representation of the experimental design. Mice were exposed to three Proton ABI dose levels 14, 16, and 18 Gy. ABI was delivered at two FLASH (HDR: 57.14–64.29 and LDR: 43.21–49.38 Gy/s) and one CONV (0.5–1.0 Gy/s) dose rates. In response to 14 Gy ABI Kaplan–Meier survival curves (C,E) and longitudinal body weight (D,F) of mice demonstrated no differences in Proton FLASH (dose‐rate: 57.14–64.29 and 43.21–49.38 Gy/s) vs. Proton CONV (dose‐rate: 0.5–1.0 Gy/s). In response to 16 Gy ABI both Proton FLASH dose‐rate (57.14–64.29 and 43.21–49.38 Gy/s) demonstrated improved survival (90% and 60%) (*p* = 0.042, *p* = 0.0793) (G,I) and body weight (H,J) compared to Proton CONV (dose‐rate: 0.5–1.0 Gy/s) irradiated mice. Exposure to 18 Gy ABI, Proton CONV irradiation (dose‐rate: 0.5–1.0 Gy/s) which resulted in 100% mortality compared to improved survival with Proton FLASH (dose‐rate: 57.14–64.29 and 43.21–49.38 Gy/s) (50% and 40% survival, respectively) (*p* = 0.013, *p* = 0.022) (K,M). Body weight gain was not comparable due to 100% lethality in Proton CONV exposure with 10 days (L,N). Data are presented as Mean ± SD; with Significant; *p < 0.05, **p < 0.005, *n* = 10 mice per group.

To evaluate the therapeutic advantage of Proton FLASH over Proton CONV irradiation in sparing normal intestinal epithelium, a survival study was performed in a mouse model of radiation‐induced intestinal injury [3, 4]. Mice were subjected to abdominal irradiation (ABI) at three different doses: 14, 16, and 18 Gy. Proton FLASH was delivered with, two dose rates consisting of low dose rate (LDR) (43.21–49.38 Gy/s) and high dose rate (HDR) (57.14–64.29 Gy/s). Proton CONV irradiation was delivered at a standard dose rate of 0.5–1.0 Gy/s (Figure 1A,B). Survival and body weight were monitored over 40 days to analyze the acute toxicity and post‐irradiation recovery.

At 14 Gy, Proton FLASH (dose‐rate: 57.14–64.29 and 43.21–49.38 Gy/s) and Proton CONV (dose‐rate: 0.5–1.0 Gy/s) irradiation demonstrated 100% survival, indicating minimal toxicity induced by the 14 Gy dose regardless of dose rate (Figure 1C,E). Consistent with the survival data, there were no significant differences in body weight between the groups (Figure 1D,F).

At a radiation dose of 16 Gy, the survival advantage of Proton FLASH over Proton CONV irradiation became distinctly evident. Under 57.14–64.29 Gy/s. dose‐rate conditions, mice irradiated with Proton FLASH exhibited a significantly improved survival rate of 90%, compared to only 40% in the Proton CONV(dose‐rate: 0.5–1.0 Gy/s) group (Figure 1G). A similar trend was observed under dose‐rate 43.21–49.38 Gy/s conditions, where Proton FLASH showed 60% survival, compared to 20% survival with Proton CONV(dose‐rate: 0.5–1.0 Gy/s) irradiation (Figure 1I). The body weight analysis further supported these findings, as mice irradiated with Proton FLASH (dose‐rate: 57.14–64.29 Gy/s) demonstrated more rapid recovery of body weight compared to those exposed to Proton CONV (dose‐rate: 0.5–1.0 Gy/s) irradiation. The average body weight gain was significantly higher in mice irradiated with Proton FLASH after 30 days of irradiation, indicating enhanced post‐irradiation recovery (Figure 1H).

At the highest irradiation dose of 18 Gy, the survival benefit of Proton FLASH irradiation (dose‐rate: 57.14–64.29 and 43.21–49.38 Gy/s) was even more pronounced. Proton CONV irradiation (dose‐rate: 0.5–1.0 Gy/s) resulted in 100% mortality (Figure 1K,M). In contrast, Proton FLASH irradiation demonstrated 50% and 40% survival rates under both the dose rates, respectively (dose‐rate: 57.14–64.29 and 43.21–49.38 Gy/sec.) (Figure 1K,M). These findings suggest that Proton FLASH irradiation reduces radiation‐induced lethality in mice models of abdominal irradiation.

### 2.2 Proton FLASH Irradiation Spares Intestinal Epithelial Morphology Compared to Proton CONV

Given the tissue‐specific sensitivity of the gastrointestinal (GI) tract to radiation‐induced injury, this study next investigated intestinal epithelial morphology in response to Proton FLASH vs. Proton CONV irradiation.

**FIGURE 2:**
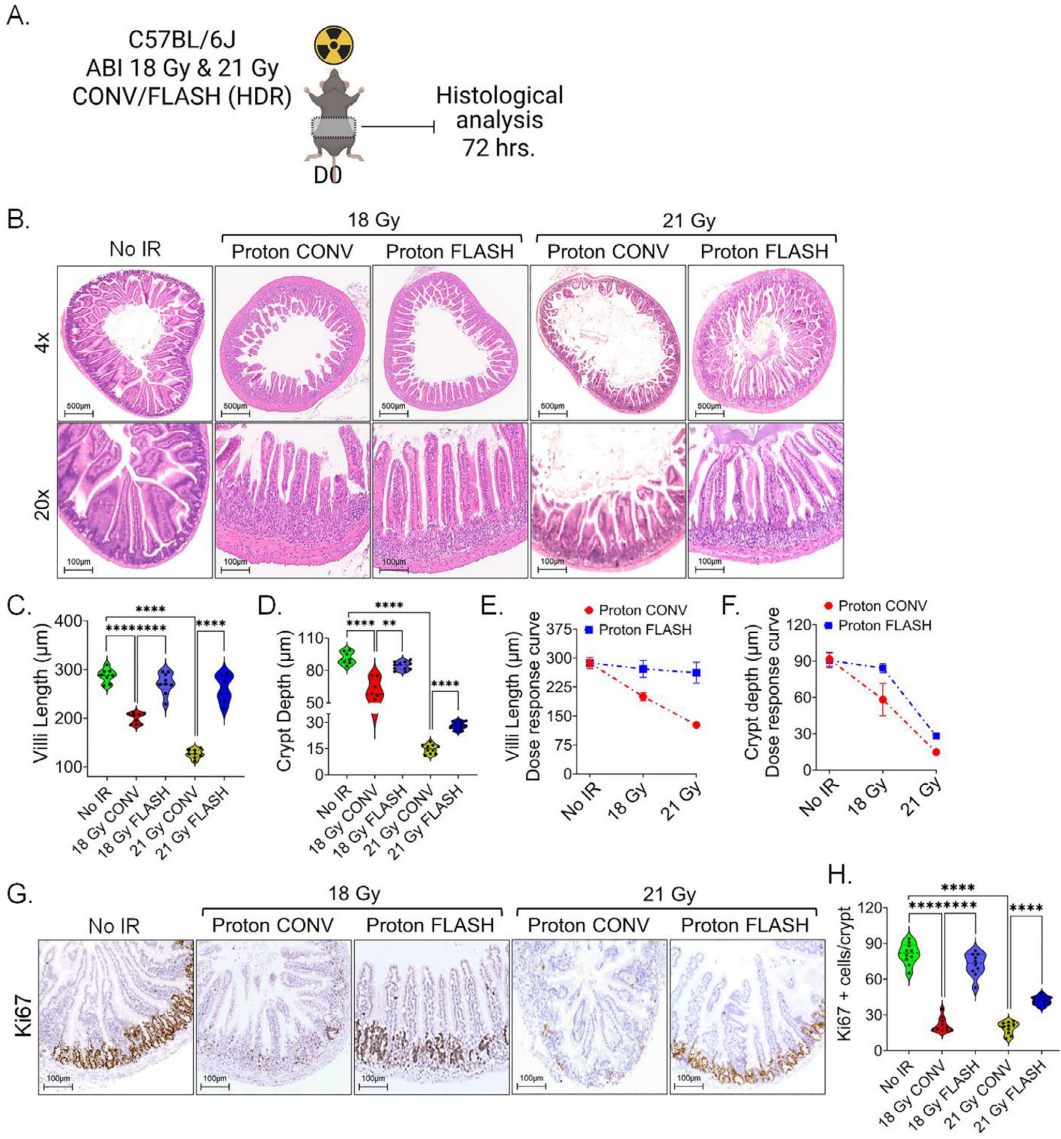
Proton FLASH irradiation preserves intestinal morphology following high‐dose ABI. (A) Schematic representation of experimental design. Mice received 18 and 21 Gy abdominal irradiation using either Proton CONV (dose‐rate: 0.5–1.0 Gy/s) or Proton FLASH (dose‐rate 57.14–64.29 Gy/s) irradiation, followed by histological analysis of jejunal sections at 72 h post‐irradiation. (B) Representative H&E‐stained jejunal sections (Transverse sections: top panel and longitudinal sections: Bottom panel) from Proton CONV (dose‐rate: 0.5–1.0 Gy/s) and Proton FLASH (dose‐rate 57.14–64.29 Gy/s) irradiated mice showed improved mucosal preservation in Proton FLASH‐irradiated groups at both 18 and 21 Gy of irradiation. (C,D) Violin plot demonstrates higher villus length and crypt depth in Proton FLASH‐irradiated mice at both 18 and 21 Gy of irradiation compared to Proton CONV dose rate. (E,F) Dose‐response curves demonstrated less decline in villus length and crypt depth with increasing dose from 18 to 21 Gy in Proton FLASH compared to Proton CONV dose rate. (G,H) showing the number of Ki67+ proliferative cells per crypt. Proton FLASH‐irradiated mice demonstrate significantly higher Ki67+ cell counts compared to Proton CONV‐irradiated animals at both 18 and 21 Gy of irradiation. Data presented as mean ± SD, with Significant; **p < 0.005, ****p < 0.00 005. *n* = 10 mice per group.

To assess the impact of high‐dose (18–21 Gy) ABI on intestinal epithelium, histological analyses of jejunal sections were conducted 72 h post‐irradiation with Proton FLASH either dose‐rate: 57.14–64.29 Gy/s (Figure 2A) or 43.21–49.38 Gy/s (Figure S1A) or Proton CONV irradiation (dose‐rate: 0.5–1.0 Gy/s) (Figure 2A). Histological evaluation revealed that Proton FLASH therapy significantly better‐preserved intestinal morphology under both 18 and 21 Gy doses with dose‐rate: 57.14–64.29 Gy/s and at 18 Gy with dose‐rate 43.21–49.38 Gy/sec compared to Proton CONV (dose‐rate: 0.5–1.0 Gy/s) irradiation (Figure 2B and Figure S1B).

Tissues from Proton FLASH‐exposed mice exhibited well‐defined villi and intact crypt structures, demonstrating maintained mucosal integrity. In contrast, Proton CONV‐irradiated intestinal tissue showed severe radiation‐induced damage, including blunted villi, crypt depletion, and mucosal flattening, particularly at 21 Gy (Figure 2B). Quantitative morphometric analysis further supported these observations, at both 18 and 21 Gy with dose‐rate: 57.14–64.29 Gy/s, Proton FLASH‐irradiated mice showed significantly greater villus length and crypt depth compared to Proton CONV‐irradiated mice (Figure 2C,D). Dose‐response curves showed a clear decline in villus length and crypt depth with increasing dose from 18 to 21 Gy, but Proton FLASH irradiation consistently reduced this decline relative to Proton CONV irradiation (Figure 2E,F).

At 18 Gy with dose‐rate 43.21–49.38 Gy/s, Proton FLASH irradiation also significantly improved both villus length and crypt depth relative to Proton CONV irradiation, demonstrating that its protective effects extend across different dose rates (Figure S1C,D).

TUNEL staining of jejunal sections at 24 h post‐irradiation (21 Gy) revealed a significant increase in intestinal epithelial apoptosis following Proton CONV irradiation. In contrast, mice exposed to Proton FLASH irradiation exhibited significantly fewer TUNEL‐positive epithelial cells, further supporting the observed sparing effect on crypt‐villus morphology in these animals (Figure S2A,B).

To further assess epithelial regenerative potential, Ki67 immunohistochemistry was used to quantify proliferating crypt cells. Proton FLASH‐irradiated mice with both the dose‐rate 57.14–64.29 and 43.21–49.38 Gy/s, showed a markedly higher number of Ki67+ cells per crypt compared to Proton CONV‐irradiated mice (*p* < 0.00 005) (Figure 2G,H and Figure S1B,E). While a dose‐dependent decline in Ki67+ cell numbers was observed, Proton FLASH‐irradiated mice retained significantly greater proliferative capacity than those receiving Proton CONV irradiation (Figure S1F)

The dose‐response curve for Ki67+ cells with Proton FLASH dose‐rate 57.14–64.29 Gy/s confirmed this pattern, Proton FLASH irradiation preserves the crypt proliferation more effectively compared to Proton CONV irradiation (Figure S1F). Collectively, these results provide strong histopathological evidence that Proton FLASH irradiation is more effective than Proton CONV irradiation in sparing intestinal epithelium from radiation toxicity.

Previous reports suggested that radiation‐induced damage in intestinal CD31+ endothelial cells influence crypt epithelial cell apoptosis [17]. In the present study mouse jejunal sections subjected to immunohistochemistry with CD31 antibody demonstrated a significant loss of endothelial cells with Proton CONV dose‐rate: 0.5–1.0 Gy/s (at 21 Gy). However, Proton FLASH (dose‐rate 57.14–64.29 Gy/s) irradiation significantly spares the CD31+ endothelial cell population within the crypt/villous region (Figure S2C,D).

Since epithelial barrier disruption permits translocation of luminal endotoxin and triggers innate immune activation [18], circulating LPS‐binding protein (LPS‐BP) and soluble CD14 (sCD14) were assessed as markers of endotoxin‐associated inflammatory signaling and epithelial barrier disruption [19, 20, 21]. Consistent with the intestinal epithelial restitution, plasma from Proton FLASH irradiated (21 Gy) mice demonstrated significantly lower levels of LPS‐BP and sCD14, suggesting preserved epithelial barrier integrity and minimal endotoxin translocation with reduced inflammatory signaling (Figure S3A,B). In contrast, Proton CONV irradiation elevated plasma levels of both LPS‐BP and sCD14, suggesting that epithelial barrier disruption subsequently enhanced endotoxin translocation.

Further, Alcian blue staining of jejunal sections demonstrates loss of goblet cells following Proton CONV irradiation, compared to Proton FLASH irradiation. The loss of goblet cells indicating damaged epithelial layer and compromised mucin production in Proton CONV irradiated tissue (Figure S3C,D).

Additionally, we performed immunofluorescence staining of epithelial tight junction proteins (ZO‐1 and Claudin‐1) and goblet cell‐derived Muc‐2 expression to assess epithelial barrier integrity and mucus layer preservation following irradiation. Proton FLASH irradiation (dose‐rate 57.14–64.29 Gy/s) preserves the epithelial tight junction proteins ZO‐1 (Figure S4A,B), Claudin‐1 (Figure S5A,B) and goblet cell‐derived Muc‐2 expression (Figure S6A,B), indicating maintained intestinal barrier integrity. However, marked loss of intestinal epithelial barrier markers was observed following Proton CONV irradiation.

Collectively, these data demonstrate that Proton FLASH irradiation spares the intestinal epithelium from radiation‐induced damage, preserves the epithelial junctional proteins and maintains mucosal barrier function.

### 2.3 Proton FLASH Preserves the Intestinal Stem Cells

**FIGURE 3:**
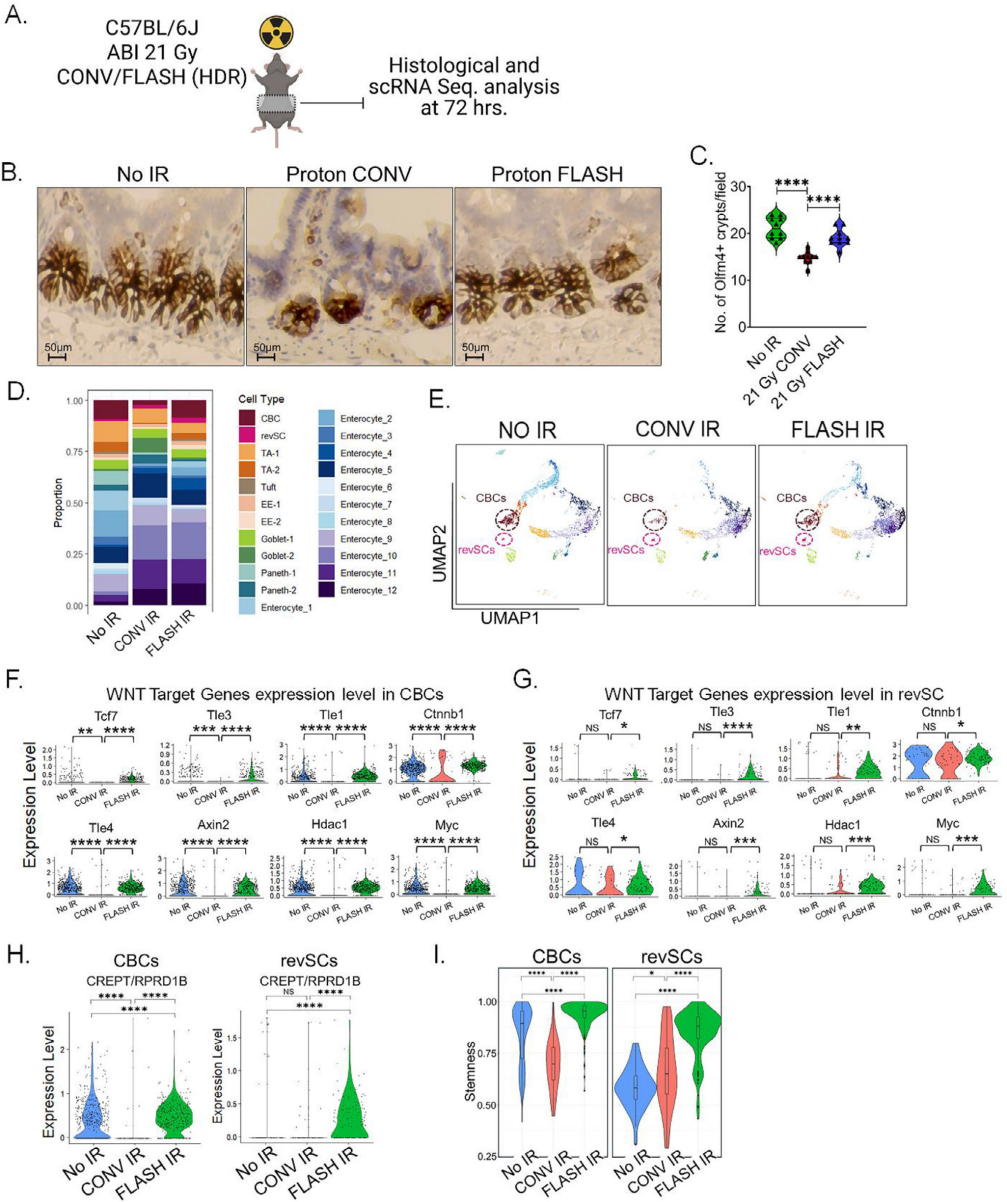
Proton FLASH spares intestinal stem cell population following high‐dose abdominal irradiation. (A) Schematic representation of experimental design. Mice received 21 Gy abdominal irradiation using either Proton CONV (dose‐rate: 0.5–1.0 Gy/s) or Proton FLASH (dose‐rate 57.14–64.29 Gy/s) irradiation, followed by histological analysis of jejunal sections and scRNA seq. analysis of intestinal epithelial cells at 72 h post‐irradiation. (B) Representative Olfm4‐stained jejunal sections from Proton CONV‐ and Proton FLASH‐irradiated mice with dose‐rate: 0.5–1.0 and dose‐rate 57.14–64.29 Gy/s, respectively. (C) Violin plot demonstrates significantly higher Olfm4+ crypts per field in Proton FLASH‐irradiated mice exposed to 21 Gy of irradiation compared to Proton CONV dose rate. (D) Bar plot showing the proportional representation of each intestinal epithelial cell type analyzed by scRNA seq in No IR, Proton CONV IR, and Proton FLASH IR groups. Higher presence of crypt base columnar cells (CBCs) and revival stem cells (revSCs) was noted in Proton FLASH IR group compared to Proton CONV IR. (E) UMAP plot representing epithelial cell‐specific cluster distribution in: No IR, Proton CONV IR, and Proton FLASH IR. Enrichment in cluster representing CBCs and revSCs population was noted. (F,G) violin plot demonstrated higher Wnt target genes expression levels in CBCs (F) and in revSCs (G) in Proton FLASH IR compared to Proton CONV IR group. (H,I) Violin plot demonstrated higher CREPT/RPRD1B expression level and stemness score in Proton FLASH irradiated CBCs and revSCs group compared to Proton CONV IR group. (Significant; *p < 0.05, **p < 0.005, ***p < 0.0005, ****p < 0.00 005, NS: not significant). *n* = 3 mice per group.

The regenerative response of the intestinal epithelium primarily depends on CBCs that express markers including *Lgr5*, *Olfm4*, *Lrig1*, and *Ascl2*. [3, 4, 22, 23]. Intestinal crypts also contain Bmi1+ve ISCs that are long‐lived, label‐retaining stem cells present at the +4 position of the crypt base. These Bmi1+ve ISCs interconvert with more rapidly proliferating LGR5+ve stem cells. Immunohistochemistry of mouse jejunal sections to detect Olmf4+ve CBCs clearly demonstrated significantly lower presence of ISCs at 72 h following 21 Gy of Proton CONV (dose‐rate: 0.5–1.0 Gy/s) irradiation exposure compared to non‐irradiated control. However, Proton FLASH exposure (at dose‐rate 57.14–64.29 Gy/s) minimizes radiation‐induced loss of CBCs and therefore supports epithelial regeneration (Figure 3A–C). Moreover, qPCR analysis of intestinal epithelial total RNA demonstrated a significant decrease in Bmi1 expression with Proton CONV exposure, compared to non‐irradiated mice. However, exposure to Proton FLASH improved Bmi expression at the level of non‐irradiated mice (Figure S7). These results clearly suggest that Proton FLASH exposure spares both Bmi1+ve quiescent and actively self‐renewing CBCs.

Previous reports suggested that lethal dose of radiation results in a complete loss of CBCs within 72–96 h post‐irradiation [23, 24] and thereby impairs the epithelial regenerative process. We therefore also examined the effect of irradiation on CBCs at 96 h post exposure. Lgr5‐EGFP‐CreERT2; R26‐ACTB‐tdTomato‐EGFP reporter mice were exposed to 21 Gy ABI with Proton FLASH (dose‐rate 57.14–64.29 Gy/s) and/or Proton CONV (dose‐rate: 0.5–1.0 Gy/s) and intestinal tissues were isolated 96 h post‐irradiation for analysis (Figure S8A). Confocal imaging of jejunal sections showed a marked depletion of Lgr5+ ISCs in Proton CONV‐irradiated mice, characterized by a significant reduction in GFP+ (Green fluorescent Protein) crypts per field (Figure S8B,C) when compared to non‐irradiated and FLASH‐irradiated mice (*p* < 0.0005 and *p* < 0.005, respectively). No significant difference was found in the number of Lgr5+ crypts per field in Proton FLASH‐irradiated mice compared to non‐irradiated mice, suggesting preservation of the functional regenerative niche (Figure S8B,C). Proton FLASH mediated sparing of ISCs and thereby maintenance of regenerative response was also evident with the presence of Ki67+ve proliferating epithelial cells even at 96 h post ABI. However, Proton CONV irradiation results in absence of Ki67 positive epithelial cells due to absence of regenerative ISCs (Figure S9A,B).

These findings suggest that, unlike Proton CONV irradiation, Proton FLASH irradiation spares the regenerative intestinal stem cell pool that is critical for intestinal epithelial regeneration following high‐dose irradiation.

### 2.4 Proton FLASH Spares Injury‐Induced Revival Stem Cells (revSCs) and Other Intestinal Epithelial Cell Population

To investigate the regeneration and differentiation dynamics of the intestinal epithelium after irradiation, single‐cell RNA sequencing (scRNA‐seq) was used to characterize epithelial cell populations. This was performed on small intestinal cells isolated 72 h after 21 Gy of ABI using Proton CONV‐irradiated, Proton‐FLASH‐irradiated, and non‐irradiated mice (Figure 3A). Single‐cell analysis of the intestinal epithelium revealed 24 distinct epithelial subpopulations, indicative of specialized lineages and varying maturation stages. Initial clustering of integrated samples established a comprehensive dataset of mouse intestinal cells. Epithelial cells, identified by Epcam expression, were then isolated and re‐integrated for further analysis (Figure S10A,B). Subsequent clustering revealed 24 clusters with unique gene expression profiles that defined functional identities (Figure S10C) of different epithelial cell types. Actively cycling CBC stem cells (marked by Smoc2, Lgr5, and Ascl2 expression) [22, 25] and injury‐induced revival stem cells (revSCs) (characterized by expression of Ly6a, Clu, Areg, and Anxa2), have previously been linked to tissue regeneration following injury [26, 27]. Proportional bar plots and UMAP plot showing the absolute number of cells demonstrated that Proton CONV irradiation depleted CBCs and revSCs (*p* = 0.2546) compared to Proton FLASH irradiation (Figure 3D,E). In contrast, Proton FLASH irradiation preserved CBCs at levels comparable to non‐irradiated controls (*p* = 0.9108) and significantly increased revSCs (*p* = 0.0017). Collectively, these findings indicate that Proton FLASH preserves ISCs and a diverse functional pool of regenerating epithelial progenitors, thereby maintaining crypt‐villus architecture post‐irradiation.

### 2.5 Enhanced Wnt Signaling in ISC Populations Following Proton FLASH Irradiation

Wnt signaling plays an important role in maintaining ISCs homeostasis, proliferation, and regeneration for epithelial renewal following radiation‐induced injury [22, 28]. To investigate whether Proton FLASH irradiation preserves or enhances Wnt signaling in ISCs compared to Proton CONV irradiation, we examined the expression of Wnt target genes in CBCs and revSCs. In both CBCs and revSCs Proton FLASH‐irradiated cells demonstrated significantly higher expression of canonical Wnt target genes including *Axin2*, *Myc*, *Tcf7*, and *Ctnnb1* compared to Proton CONV‐irradiation (Figure 3F,G). Additionally, the upstream transcriptional regulator *Tcf7* in CBCs, while *Tcf7*, *Tcf7l2* in revSCs were highly expressed in Proton FLASH‐irradiated cells compared to Proton CONV‐irradiated cells suggesting enhanced Wnt/β‐catenin‐dependent transcriptional activity within the ISCs. However, the transcription factor corepressors like Transducing‐like enhancer of split proteins (*TLEs*: *Tle1*, *Tle3*, and *Tle4)* [29, 30] were also highly expressed in Proton FLASH‐irradiated cells compared to Proton CONV‐irradiated cells, indicating that homeostatic balance of Wnt signaling is maintained with overall pathway activation [31].

These findings suggest that Proton FLASH irradiation not only spares but actively supports the regenerative capacity of ISCs by enhancing Wnt signaling compared to Proton CONV irradiation.

To further explore the downstream effects of enhanced Wnt/β‐catenin signaling in Proton FLASH‐irradiated ISCs, we assessed the expression of CREPT/RPRD1B (Cell‐cycle Related and Expression‐elevated Protein in Tumor), a transcriptional co‐regulator known to promote cell proliferation by enhancing Wnt/β‐catenin signaling [32]. CREPT/RPRD1B has been involved in supporting epithelial regeneration by enhancing ISCs regenerative capacity and maintaining stem cell identity following radiation injury [33, 34]. Radiation‐induced injuries often impair stemness of intestinal epithelial cells. To evaluate the impact of Proton FLASH irradiation on regenerative potential of ISCs, we analyzed CREPT/RPRD1B expression (Figure S10D) and stemness scores measured by CytoTRACE algorithm [35] (Figure S10E) in CBCs and revSCs. CREPT/RPRD1B expression was significantly higher in both CBCs and revSCs of Proton FLASH‐irradiated cells compared to Proton CONV‐irradiated cells (Figure 3H). Remarkably, both CBCs and revSCs also demonstrated significantly higher stemness scores in Proton FLASH‐irradiated cells, which suggest a preservation or enhancement of their stem cell identity (Figure 3I). In contrast, Proton CONV irradiation led to a significant reduction in stemness in both CBCs and revSCs (Figure 3I). These results further indicate that Proton FLASH irradiation promotes CREPT/RPRD1B expression and sustains stemness in ISCs populations, which potentially contribute to epithelial regeneration and reduced radiation‐induced toxicity.

To predict the potential signaling pathways involved in the Proton FLASH‐mediated sparing effect in ISCs, a pathway enrichment analysis was performed based on scRNA seq gene expression data in revSCs and CBCs. Both revSCs and CBCs demonstrated enrichment of genes related to repair and regenerative pathways.

**FIGURE 4:**
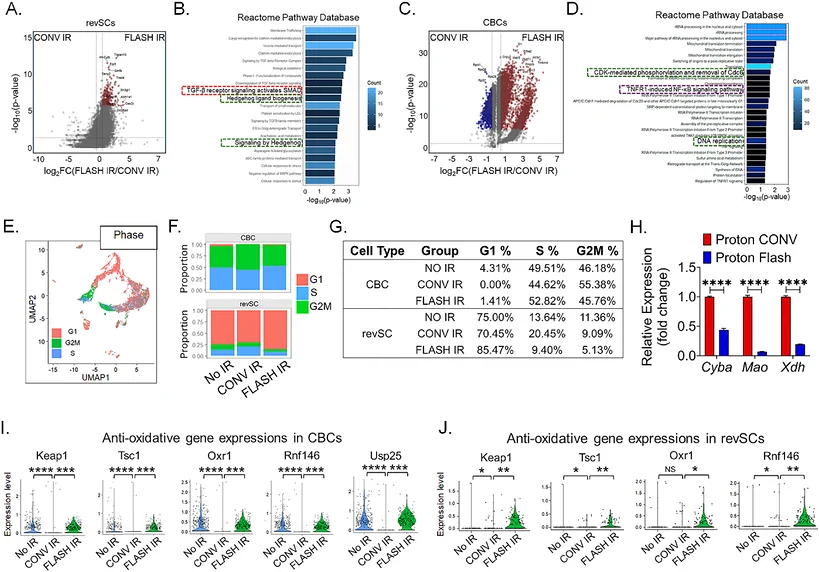
Proton FLASH irradiation demonstrated enrichment of genes associated with regenerative pathways, cell cycle and oxidative stress in CBCs and revSCs. (A,B) Pathways enrichment analysis based on a Reactome pathway database demonstrated enrichment of genes associated with TGF‐β receptor/SMAD signaling, Hedgehog signaling, and other epithelial repair pathways in revSCs. (C,D) Pathways enrichment analysis based on a Reactome pathway database demonstrated enrichment of genes associated with CDK‐mediated phosphorylation and removal of Cdc6, TNFR1‐induced NF‐κB signaling and DNA replication pathways in CBCs. (E) UMAP plot showing cell‐cycle phase distribution (G1, S, G2/M) across epithelial cells. (F) Bar diagram demonstrating quantification of cell cycle phases (G1, S, G2/M) within CBC and, revSC clusters across different groups. (G) Table demonstrating cell cycle phase distribution of selected intestinal epithelial cell types (CBC, revSC). (H) qPCR analysis demonstrated, low expression of oxidative stress‐related gene expression in Proton FLASH irradiated intestinal epithelial cells compared to Proton CONV irradiated cells. (I,J) scRNA‐seq analysis showing significant upregulation of antioxidant genes (*Keap1, Usp25, Tsc1, Oxr1, Rnf146*) in CBCs and revSCs following Proton FLASH irradiation, indicating enhanced oxidative stress defense. (Significant; *p < 0.05, **p < 0.005, ***p < 0.0005, ****p < 0.00005, NS: not significant), *n* = 3 mice per group.

The revSCs population demonstrated genes associated with TGF‐β receptor signaling and Hedgehog signaling pathway (Figure 4A,B). According to previous reports, TGF‐β signaling is involved in ISC proliferation and differentiation and has been implicated in protecting the intestinal barrier during injury repair [36]. Hedgehog signaling is known to support intestinal regeneration by influencing mesenchymal niche activity and maintaining epithelial architecture [37].

Analysis of CBCs highlighted genes associated with CDK‐mediated phosphorylation and removal of Cdc6 as well as DNA replication pathways (Figure 4C,D). These pathways regulate the G1/S phase transition and initiation of DNA replication [38, 39]. Genes related to TNFR1‐induced NF‐κB signaling pathways involved in promoting intestinal epithelial cell survival [40, 41] were also enriched in Proton FLASH‐treated CBCs. However, considering the differences in ISCs population in Proton CONV and Proton FLASH irradiation, further molecular and functional validation is needed to establish the involvement of any of these signaling pathways in the sparing effect of Proton FLASH on ISCs.

### 2.6 Proton FLASH Regulates Expression of Cell Cycle Related Genes in ISCs

Analysis of cell cycle‐related genes in CBCs and revSC suggested that Proton FLASH exposure delayed the cell cycle progression post exposure compared to Proton CONV exposure. At 72 h post Proton CONV exposure, a higher percentage of CBCs progressed to G2/M phase (55.38%) compared to Proton FLASH (45.76%) (Figure 4E–G). Similar response was also observed in revSCs. Due to quiescence and slow cycling nature, a majority of the revSCs were observed in the G1 phase and a minimal number of cells progressed to G2/M phase (Figure 4E–G). However, revSCs with Proton CONV exposure had a higher percentage of cells in S and G2/M phase (Figure 4G). It has been reported that transient cell cycle arrest and thereby delay in cell cycle progression immediately following radiation in ISCs helps to reduce DNA damage [42]. Our data further suggested that delay in progression of the cell cycle following Proton FLASH exposure may contribute to preservation of CBCs and revSCs compared to Proton CONV exposure.

Collectively, these findings suggest that Proton FLASH irradiation triggers a distinct transcriptional program in CBCs that enhances cell survival, delayed re‐entry into the cell cycle and promotes regenerative signaling and activation of revSCs, all of which enables more efficient regeneration of the intestinal epithelium compared to Proton CONV irradiation.

### 2.7 Proton FLASH Exposure Modulates Oxidative Stress in ISCs

It has been reported that the normal‐tissue sparing effects of FLASH‐RT is due to the rapid reduction of intracellular oxygen, namely radiolytic oxygen depletion (ROD) [8, 43]. Oxygen increases the radiation susceptibility of cells with a maximum Oxygen Enhancement Ratio (OER) between 2.5 and 3.5 [44]. Compared to CONV RT, FLASH RT dose rate promote depletion of O_2_ critical to the biological response due to competition between the depletion rate and the re‐diffusion rate of O_2_ from the capillaries [44]. Proton FLASH may increase the probability of radical–radical recombination and therefore minimize the free radicals toxicity, such as oxidative stressdue to transient accumulation of a large concentration of radical species during a short time window [45]. In a mouse model of abdominal irradiation, we observed reduced expression of oxidative stress genes such as *Cyba, Mao, Xdh* [46] (Figure 4H) in intestinal epithelium with exposure to Proton FLASH compared to Proton CONV at 72 h post exposure. scRNA seq analysis demonstrated significant upregulation of multiple anti‐oxidative gene expressions such as *Keap1, Usp25, Tsc1, Oxr1, Rnf146* in CBCs (Figure 4I) and revSCs (Figure 4J) in response to Proton FLASH irradiation compared to Proton CONV irradiation.

Modulation of radiation‐induced oxidative stress in response to Proton FLASH was further validated by immunofluorescence staining of total OXPHOS protein in jejunal section of these irradiated mice. Total OXPHOS protein immunostaining detects a complex of multiple subunits of the respiratory chain complex crucial for oxidative phosphorylation, such as Complex I (NDUFB8), II (SDHB), Complex III (UQCRC2) IV (COXII), and Complex V (ATP5A) subunits) which together constitute the mitochondrial respiratory chain embedded within the inner mitochondrial membrane [47]. As a result, the immunofluorescence signal reflects the overall abundance and integrity of the mitochondrial OXPHOS system rather than individual complex‐specific expression. Proton CONV irradiation caused a significant increase in total OXPHOS protein levels (*p* < 0.005) compared to Proton FLASH irradiation, whereas Proton FLASH irradiated mouse intestinal tissue maintained total OXPHOS expression at levels comparable to non‐irradiated controls (Figure S11A,B). Moreover, Dihydroethidium (DHE) staining, which specifically detects superoxide anion (O_2_•−) [48], demonstrated significantly less superoxide‐associated reactive oxygen species (ROS) levels in jejunal sections at 24 h following Proton FLASH compared with Proton CONV irradiation (Figure S12A,B). ROS such as hydroxyl radicals, superoxide, hydrogen peroxide, are highly reactive molecules that damage DNA by oxidizing bases and breaking the sugar backbone [49]. In the present study, DNA damage was analyzed in the irradiated tissue using γH2AX immunohistochemistry [50]. At 24 h, Proton CONV irradiation induced a substantial increase in γH2AX‐positive cells per crypt, whereas Proton FLASH irradiated crypts showed significantly fewer γH2AX‐positive cells, indicating a reduction in double‐strand DNA breaks (Figure S13A,B).

These findings suggest a dual mechanism for the tissue‐sparing effects of Proton FLASH irradiation in the murine intestine: the mitigation of injury‐related stress responses and the simultaneous preservation of regenerative signaling.

### 2.8 Proton FLASH Irradiation Preserves Gut Microbiome α‐Diversity and Enriches Commensal Bacterial Composition

**FIGURE 5:**
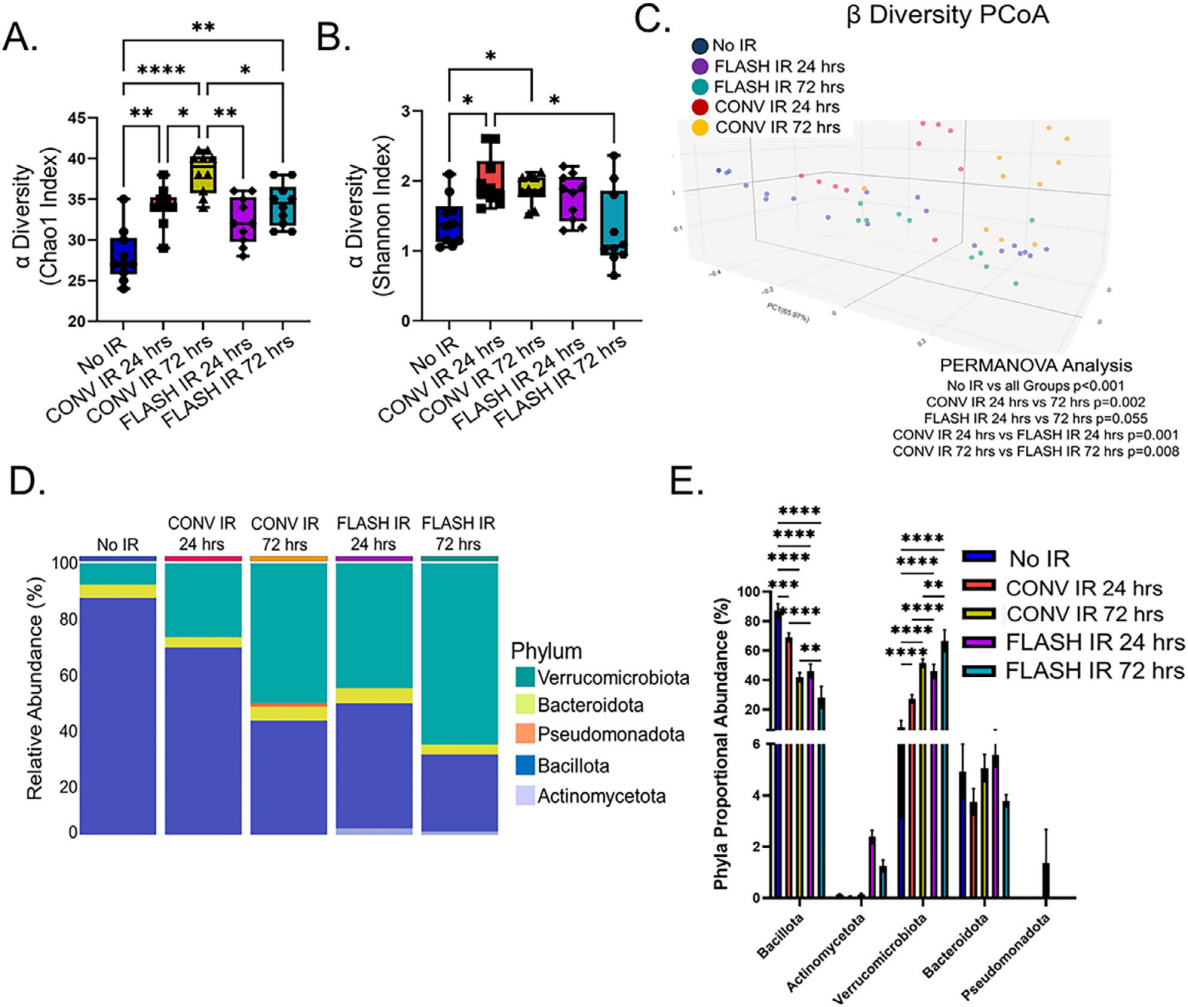
Proton FLASH irradiation preserves gut microbial *α*‐diversity compared to Proton CONV irradiation. (A) Alpha diversity analysis (Chao1 index) showing that all Proton CONV‐irradiated groups exhibited increased richness relative to non‐irradiated controls. Proton FLASH‐irradiated mice maintained significantly higher diversity only at 72 h post‐irradiation compared to controls, but α‐diversity was significantly decreased relative to Proton CONV at the same timepoint. (B) Shannon α‐diversity index demonstrates that Proton CONV, but not Proton FLASH, irradiation resulted in elevated α‐diversity. (C) β‐ diversity analysis using Bray–Curtis Principal Coordinate Analysis (PCoA) shows distinct clustering of microbial communities between Proton FLASH and Proton CONV irradiated groups, with PERMANOVA confirming statistically significant differences. (D,E) Phylum‐level taxonomic profiling indicating that both irradiation modalities reduced Bacillota abundance; however, Proton CONV irradiation caused a marked increase in Bacillota relative to Proton FLASH at matched timepoints, whereas Proton FLASH maintained relatively higher Verrucomicrobiota and lower Bacillota at both 24 and 72 h post‐irradiation compared to Proton CONV. Data are shown as mean ± SD (Significant; *p < 0.05, **p < 0.005, ****p < 0.00 005), *n* = 10 mice per group.

Proton FLASH irradiation preserves gut microbial *α‐*diversity more effectively than CONV irradiation, although differences in β‐diversity community composition remain significant compared to non‐irradiated controls. To evaluate the impact of irradiation dose on the intestinal microbiome, we analyzed fecal samples collected from mice irradiated with either Proton FLASH or CONV irradiation. Alpha diversity analysis, using Chao1 index, revealed that all CONV irradiated groups demonstrated increased richness relative to non‐irradiated controls. FLASH‐irradiated mice maintained significantly higher microbial α‐diversity compared to the non‐irradiated controls, only at 72 h post‐irradiation and when compared with CONV irradiation at 72 h was significantly decreased (Figure 5A). Shannon α‐diversity index measurement indicates CONV irradiation, but not FLASH, resulted in elevated α‐diversity (Figure 5B). β‐diversity analysis using Bray–Curtis Principal Coordinate Analysis (PCoA) demonstrated a distinct clustering of microbial community between FLASH and CONV irradiated groups. Further the PERMANOVA analysis confirmed statistically significant differences between these distinct clustering (Figure 5C), which suggests that the Proton FLASH and Proton CONV induce markedly different effects on gut microbial composition. Phyla taxonomic profiling showed that both radiation modalities reduced Bacillota phyla proportional abundance. However, Proton CONV irradiation resulted in a higher proportional abundance when compared with Proton FLASH irradiation at matching timepoints (Figure 5D,E), whereas Proton FLASH IR displayed higher proportional abundance of Verrucomicrobiota and lower Bacillota (Figure 5D,E). Both Proton CONV and Proton FLASH irradiation resulted in an increased Verrucomicrobiota abundance when compared to non‐irradiated controls. At 24 and 72 h post irradiation, Proton FLASH led to significantly higher proportional abundance of Verrucomicrobiota than Proton CONV (Figure 5D,E). These findings suggest that both modalities of irradiation shift the gut microbiome β‐diversity; however, Proton CONV irradiation modifies microbial α‐diversity (richness and evenness) more severely than Proton FLASH irradiation, suggesting Proton FLASH irradiation may better preserve gut‐microbial community diversity to that of non‐irradiated controls.

**FIGURE 6:**
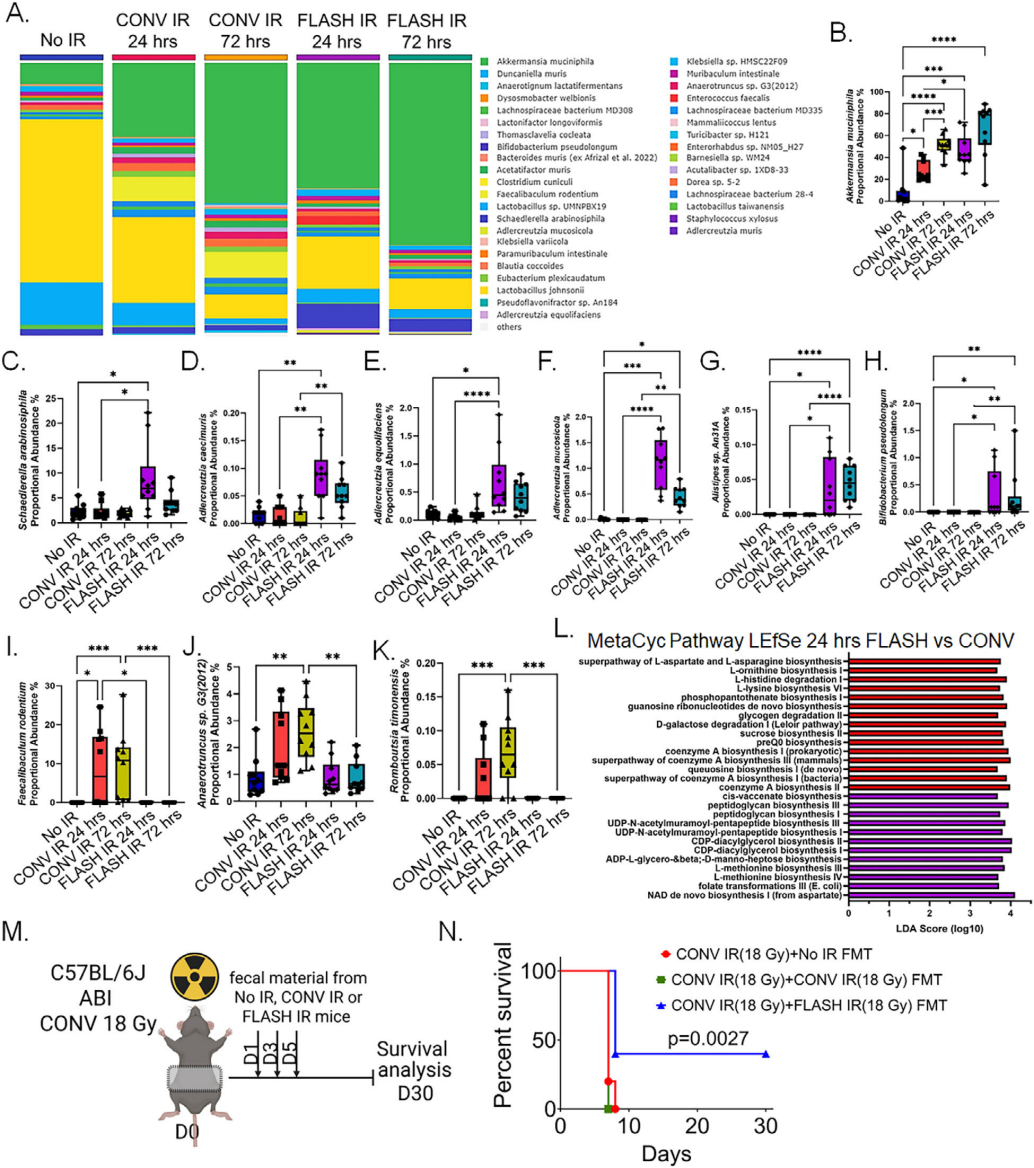
Proton FLASH irradiation preserves beneficial microbial functions. (A) Species‐level taxonomic profiling showing distinct microbial composition between groups, with notable enrichment in Proton FLASH‐irradiated mice. (B) Significant increase in *Akkermansia muciniphila* abundance at 24 h post‐Proton FLASH irradiation compared to Proton CONV, suggesting improved mucosal resistance. (C–F) Enrichment of *Schaedlerella arabinosiphila, Adlercreutzia equolifaciens, Adlercreutzia caecimuris*, and *Adlercreutzia mucosicola* in Proton FLASH mice at both the timepoints, indicating restoration of short‐chain fatty acid‐ and equol‐producing commensals that promote gut barrier integrity and anti‐inflammatory effects. (G,H) Increased abundance of *Alistipes sp. An31A and Bifidobacterium pseudolongum* in Proton FLASH mice, taxa associated with enhanced mucin production, mucus layer strengthening, and metabolic health. (I–K) Higher abundance of potentially harmful taxa (*Faecalibaculum rodentium, Anaerotruncus sp. G3(2012), Romboutsia timonensis*) in Proton CONV irradiation, with *R. timonensis* previously linked to GI distress and inflammation. (L) LEfSe analysis of MetaCyc pathways at 24 h shows significant enrichment in Proton FLASH mice of biosynthetic pathways including NAD de novo biosynthesis, L‐methionine biosynthesis, and folate transformations, suggesting enhanced metabolic potential and mucosal support. (M) Schematic representation of fecal microbiota transplantation (FMT) experimental design in which Proton CONV‐irradiated recipients received microbiota from either Proton CONV or Proton FLASH‐irradiated donor mice. (N) Survival analysis showing 100% mortality within 10 days in Proton CONV recipients of Proton CONV‐FMT, vs. 40% survival beyond 30 days in Proton CONV recipients of Proton FLASH‐FMT, indicating functional protection supported by Proton FLASH‐preserved microbiota. *n* = 5 mice per group (Significant; *p < 0.05, **p < 0.005, ***p < 0.0005, ****p < 0.00 005).

Further, the taxonomic profiling at the species level revealed a distinct microbial composition across groups, with notable enrichment of specific taxa in the Proton FLASH irradiated group (Figure 6A). Among these, *Akkermansia muciniphila* was significantly elevated in Proton FLASH irradiated group at 24 h compared to tProton CONV irradiated groups suggesting improved mucosal resistance following irradiation [51, 52] (Figure 6B). Similarly, *Schaedlerella arabinosiphila*, *Adlercreutzia equolifaciens*, *Adlercreutzia caecimuris*, and *Adlercreutzia mucosicola* were significantly enriched in Proton FLASH irradiation group at various timepoints compared to Proton CONV irradiation groups (Figure 6C–F), potentially indicating restoration of key short‐chain fatty acid and equol‐producing commensals which would help in gut barrier integrity and anti‐inflammatory properties, respectively [53, 54, 55]. *Alistipes sp. An31A*, which is associated with gut barrier function and metabolic health, and *Bifidobacterium pseudolongum* which increases the mucin production and strengthens the mucus layer [56], were both markedly increased in Proton FLASH irradiated mice compared to Proton CONV irradiation (Figure 6G,H) further supporting the enrichment of beneficial commensal taxa in Proton FLASH irradiation group. In contrast, Proton CONV radiation led to enrichment of *Faecalibaculum rodentium*, *Anaerotruncus sp. G3(2012), Romboutsia timonensis* when compared with non‐irradiated controls and Proton FLASH irradiation at various time points (Figure 6I–K). Previous literature associated GI distress and inflammation with intestinal *Romboitsia timonensis* abundance [57], highlighting microbiota disruption under Proton CONV irradiation. Furthermore, the functional microbial gene enrichment alterations, LEfSe analysis (Figure 6L) of MetaCyc pathways comparing Proton FLASH irradiation (purple bars) and Proton CONV irradiation (red bars) at 24 h revealed significant enrichment of microbial biosynthetic pathways in the Proton FLASH group, including NAD de novo biosynthesis, L‐methionine biosynthesis, and folate transformations, suggesting enhanced metabolic potential, redox balance modification [58], and mucosal support [59] in Proton FLASH irradiated mice. Collectively, these data demonstrate that Proton FLASH irradiation preserves beneficial microbial functions.

To functionally assess whether the gut microbiota from Proton FLASH‐exposed mice mitigates radiation toxicity, we conducted a fecal microbiota transplantation (FMT) experiment. The recipient mice were first irradiated to Proton CONV irradiation (dose‐rate: 0.5–1.0 Gy/s) with LD100/10 (18 Gy) dose ensuing primarily GI toxicity associated mortality [3, 4, 22, 23] and were then orally gavage with fecal material derived from donor mice that had previously irradiated with 18 Gy of irradiation either Proton CONV (dose‐rate: 0.5–1.0 Gy/s) or Proton FLASH irradiation (dose‐rate 57.14–64.29 Gy/s) (Figure 6M). Proton CONV irradiated mice receiving FMT from Proton CONV irradiated donors demonstrated 100% mortality with 10–15 days post irradiation, suggesting death from radiation‐induced gastrointestinal syndrome [3, 4, 22, 23]. However, FMT from Proton FLASH‐irradiated mice rescued Proton CONV‐irradiated recipient mice from radiation‐induced lethality and improved survival in 40% of mice beyond 30 days post irradiation (Figure 6N).

To assess the impact of FMT on the intestinal epithelium of Proton CONV irradiated recipient mice, histological analyses of jejunal sections were performed at day 4 post‐irradiation (Figure S14A). Histological evaluation revealed that FMT from Proton FLASH‐exposed mice significantly preserved intestinal morphology with significantly greater villus length and crypt depth in these recipient mice compared to FMT from Proton CONV irradiated mice (Figure S14B–D).

IHC of these jejunal sections demonstrated a higher number of Ki67+ cells per crypt in the Proton FLASH FMT‐treated mice compared to Proton CONV FMT‐treated mice (Figure S14E,F). Collectively, these findings indicate that FMT derived from Proton FLASH‐irradiated donors significantly promotes intestinal epithelial regeneration and proliferative recovery following Proton CONV irradiation.

This approach clearly suggested that Proton FLASH‐mediated enrichment of mucoprotective commensal microbiomes is beneficial to mitigate intestinal injury by preserving intestinal architecture and enhancing epithelial regenerative capacity. These findings indicate that the microbial communities preserved or enriched by Proton FLASH irradiation are functionally capable of enhancing resistance to radiation‐induced injury [60].

## 3 Discussion

Determination of the mechanism behind the tissue‐sparing effect of ultra‐high dose rate (FLASH) irradiation is important for its clinical application. In the present study, in a mouse model of radiation‐induced gastrointestinal syndrome, we have observed that Proton FLASH irradiation spares intestinal stem cell population including the revSC damage‐induced stem cell population with the better preservation of the gut microbiome population. The presence of functionally active intestinal stem cells is important to spare the mucosal epithelium from radiation injury. Proton FLASH spares the intestinal stem cells with the restitution of epithelial crypt villus architectures, maintenance of epithelial barrier function and reduction of radiation‐induced lethality in mice. Exposure to this ultra‐high dose rate also demonstrated significant reduction in radiation‐induced oxidative stress compared to Proton CONV dose rate. All this evidence clearly suggests that Proton FLASH exposure is less damaging to mucosal health compared to Proton CONV exposure.

ISC lineages consist of actively self‐renewing Olfm4+ve/Lgr5+ve CBCs [26, 61], quiescent Bmi+ve/K19+ve cells located in the +4 position [61] from the crypt base and injury‐induced revSCs located higher in crypt [26]. The functional hierarchy of these cell types tightly regulates intestinal epithelial homeostasis and regeneration. In response to genotoxic stress, such as radiation, loss of these cell types primarily depends on their radio sensitivity. Several reports from our groups clearly demonstrated that Lgr5+ve CBCs are the most radiosensitive of the ISCs [3, 4, 62]. Moreover, restitution of these CBCs from radiation toxicity is important to maintain the epithelial regenerative response. In our mouse model of radiation‐induced gastrointestinal syndrome, we observed that Proton FLASH spares CBCs which results in the maintenance of regenerative epithelium as demonstrated by Ki67+ve cells. Revival stem cells (revSCs) defined by transient induction of clusterin (CLU) expression‐rapidly expand and differentiate into multiple intestinal epithelial lineages during intestinal regeneration [34]. Therefore, presence of functional revSCs is also important to repopulate different cell types of the intestinal epithelium following injury. Proton FLASH exposure spares revSCs compared to Proton CONV dose rates and therefore supports the observed intestinal epithelial regenerative response following Proton FLASH.

Proton FLASH exposure minimizes normal tissue oxygen consumption which is similar to a hypoxic state [63]. We have observed that compared to Proton CONV dose rate, Proton FLASH exposure was associated with reduce oxidative stress and lower ROS production in intestinal epithelial cells. scRNA seq data of cell cycle markers indicated that ISCs including Lgr5+ve CBCs and revSCs are slower in cell cycle progression with Proton FLASH exposure compared to Proton CONV dose rate. At 72 h post Proton CONV exposure a higher percentage of CBCs and revSCs were in the G2/M phase compared to the Proton FLASH. Reduction in cell cycle progression may contribute to less oxygen consumption due to reduction in energy demand [64]. The relatively slower cell cycle progression observed in ISCs following Proton FLASH irradiation may also contribute to reduced susceptibility to radiation‐induced DNA damage compared to Proton CONV exposure. In summary Proton FLASH exposure is associated with reduced oxidative stress and slower cell cycle progression in ISCs.

Commensal gut microbiota is primarily anaerobic and very sensitive to oxidative stress [65, 66]. Increased oxygenation and oxidative stress in the gut, often associated with dysbiosis (an imbalance in the gut microbiota), can negatively impact the abundance and survival of these sensitive anaerobic bacteria [65, 67]. This can further disrupt gut homeostasis. Our study clearly demonstrated that Proton FLASH exposure, which results in less ROS production and oxidative stress maintains healthy gut microbiome α‐diversity compared to exposure with Proton CONV dose rate. Our findings also highlighted the prevalence of some key microbiome species such as *Akkermansia muciniphila, Alistipes sp*. critical for mucosal epithelial regeneration and maintenance of barrier function [68, 69]. On the contrary Proton exposure with CONV dose rate promotes abundance of species, such as *Romboitsia timonensis* which is associated with GI distress and inflammation [70]. In addition, our FMT study further validates that Proton FLASH irradiation exposure impacts gut microbiome populations that result in improved overall survival and limits radiation‐induced intestinal damage, favors the maintenance of functional healthy gut microbiome. FMT from Proton FLASH‐exposed mice minimized the toxicity and improved survival in mice exposed to abdominal irradiation with Proton CONV dose rate.

## 4 Conclusion

The present study established a series of evidence suggesting the beneficial role of Proton FLASH abdominal exposure to spare mucosal toxicity at a dose level which is 100% lethal in Proton CONV dose rate. Moreover, Proton FLASH modulated the mucosal redox biology in favor of intestinal stem cell survival and stability of the commensal microbiome to maintain gut homeostasis and regeneration. Importantly, this study reports for the first time the pre‐clinical use of pulsed, 228 MeV pencil beam scanning Proton FLASH irradiation, thereby providing a unique platform for evaluating biological responses to this novel radiation modality. Therefore, these findings support the potential of Proton FLASH to improve the therapeutic ratio and justify further preclinical and clinical evaluation.

## 5 Experimental Section

### 5.1 Animals

Seven to eight weeks‐old male C57BL6/J mice, Lgr5‐eGFP‐IRESCreERT2 mice, Gt (ROSA)26Sortm4(ACTB‐tdTomato‐EGFP) Luo/J mice, were purchased (The Jackson laboratory, Bar Harbor, ME, USA) for experimental studies. Lgr5‐eGFP‐IRES‐CreERT2 mice were crossed with Gt (ROSA)26Sortm4(ACTB‐tdTomato‐EGFP) Luo/J mice (Jackson Laboratories) to generate Lgr5/eGFP‐IRES‐Cre‐ERT2; R26‐ACTB‐tdTomato‐EGFP [23, 71] and In Gt (ROSA)26Sortm4(ACTB‐tdTomato‐EGFP) Luo/J mice tdTomato is constitutively expressed (independent of Cre recombination) in the membrane of all cells, and therefore allows better visualization of cellular morphology. All the animals were maintained *ad‐libitum*, and all studies were performed under the guidelines and protocols of the Institutional Animal Care and Use Committee of the University of Kansas Medical Center. Animal studies were performed under the experimental protocols approved by the Institutional Animal Care and Use Committee of the University of Kansas Medical Center (ACUP number 22‐01‐215).

### 5.2 Proton Irradiation procedure

Abdominal irradiation (ABI) was performed on mice that was anesthetized with Ketamine (87.5 mg/kg body weight) and Xylazine (12.5 mg/kg body weight) using a FLASH‐capable Proton synchrocyclotron (IBA ProteusONE) (Figure S15). This is the first study to utilize this system to deliver pulsed, 228 MeV pencil beam scanning Proton FLASH irradiation. A brass collimator was used to collimate the proton beams with a 2 × 3.5 cm^2^ opening which conferred dosimetric sparing to organs‐at‐risk (OARs) outside of the mice's gastrointestinal (GI) region. For this study, three Proton ABI dose levels (14, 16, and 18 Gy) were delivered at two FLASH (57.14–64.29 and 43.21–49.38 Gy/s) and one CONV (0.5–1.0 Gy/s) dose rates to assess the differential impact of Proton FLASH and Proton CONV irradiation survival outcomes. Post irradiation, all mice were monitored with activity levels and body weight assessed. Mice that reached a moribund status and/or experienced >20% body weight loss were considered endpoints and were euthanized.

The two different kinds of FLASH ABI fields were delivered: (1) High Uniformity/Low Ultra‐High Dose Rate (L‐UHDR) and (2) Low Uniformity/High Ultra‐High Dose Rate (H‐UHDR). H‐UHDR was an earlier Proton FLASH ABI iteration constructed from a 5 × 8 uniformly weighted proton spot map with 0.5 cm spot spacings for a total of 40 proton spots centered on the brass collimator's opening. Proton‐collimator scattering will result in an in‐scatter which slightly increases the central dose of the Proton FLASH irradiation field. GI‐HU‐UHDR is designed to result in a more uniform Proton FLASH 2D profile by extending the uniformly weighted spot‐map into the brass collimator to increase the peripheral doses relative to the central doses. GI‐HU‐UHDR consists of a 6 × 9 uniformly weighted Proton FLASH PBS spot‐map with similar spot spacings for a total of 54 Proton spots. As more spots are delivered, the corresponding Proton FLASH dose rates will be lowered for GI‐HU‐UHDR relative to GI‐LU‐UHDR. The ProteusONE synchrocyclotron outputs pulsed UHDR Protons with a pulse repetition rate of 1 kHz and all spot maps were designed with a raster‐scanning pattern to eliminate any spot‐slewing deadtimes and this was verified retrospectively with log‐file analyses. Subsequently, nominal Proton FLASH dose rates were calculated by dividing the nominal delivered Proton FLASH ABI doses by their corresponding beam delivery times, which was determined from the total number of Proton pulses delivered.

To ensure similar dose‐rates across multiple Proton FLASH ABI experiments, the number of pulses per spot was fixed. For 14, 16, and 18 Gy GI‐LU‐UHDR deliveries, 6, 7, and 7 Proton pulses per spot were delivered, respectively, which corresponded to delivery times of 0.240, 0.280, and 0.280 s, respectively, and correspondingly, Proton FLASH dose‐rates of 58.33, 57.14, and 64.29 Gy/s, respectively. The differences in the dose‐rates for the different dose levels are inevitable given the discreteness of the number of pulses delivered. For 14, 16, and 18 Gy GI‐HU‐UHDR deliveries, 6, 6, and 7 Proton pulses per spot were delivered respectively which corresponded to delivery times of 0.324, 0.324, and 0.378 s, respectively, and correspondingly, Proton FLASH dose‐rates of 43.21, 49.38, and 47.62 Gy/s, respectively. Uniformity scores were defined as the ratio of the peripheral dose at 50% of the respective axes’ field sizes to the central field dose. GI‐LU‐UHDR (HDR) uniformities in the *x*‐ and *y*‐directions were calculated to be 97.61% and 92.22%, respectively, whereas the corresponding ones for GI‐HU‐UHDR (LDR) were 98.26% and 95.50%, respectively.

### 5.3 Histology

The histological analyses jejunal sections were analyzed 72 h post‐irradiation with either LDR or HDR at 18 and 21 Gy of Proton FLASH and Proton CONV irradiation. Animals were euthanized and intestines were collected for histological analysis. The intestinal tissues were washed thoroughly by PBS to eliminate intestinal contents. Subsequently, the jejunum was fixed using 10% neutral‐buffered formalin followed by embedding in paraffin and sectioning into 5‐µm‐thick sections for hematoxylin and eosin (H&E) and immunohistochemistry (IHC) staining. All the H&E staining was performed at the Histoserve Inc. (Germantown, MD). The histopathological analysis was conducted for the assessment of crypt proliferation rate, villous length, and crypt depth to analyze the intestinal tissue morphological changes.

### 5.4 Immunohistochemistry

Jejunal tissue samples were collected and subjected to paraffin embedding followed by immunohistochemical staining for Ki67/CD31 and/or γH2AX. Paraffin‐embedded sections were deparaffinized and rehydrated through a graded series of alcohols. Before immunostaining, antigen retrieval was performed by heating slides in antigen retrieval solution Tris‐EDTA buffers (Ki67) and Sodium‐citrate (γH2AX and CD31). After antigen retrieval, sections were blocked with a peroxidase block and incubated with the primary antibody anti‐Ki67 (Cell Signaling, Cat No. 12202, 1:1500 dilution) and γH2AX (Cell Signaling, Cat No. 9718, 1:1000 dilution), ant‐CD31 (Cell Signaling, Cat No. 77699, 1:250 dilution), overnight at 4°C in t dark humidified chamber. Followed by detected with an HRP‐conjugated goat‐anti‐rabbit secondary antibody. The HRP was then visualized with enhanced diaminobenzidine (DAB). The slides were counterstained with hematoxylin, dehydrated with a graded series of alcohol, cleared in xylene, and mounted with a permanent mounting medium. Slides were scanned and imaged on an Aperio AT2 at 40X (0.25 µm/px). The identification of murine crypts within the tissue sections was employed using an established histological criterion as previously reported by Potten et al. [72]. To determine the proliferation index, Ki67‐positive nuclei were expressed as a percentage of the total number of cells per crypt, with 50 well‐defined crypts quantified per mouse. To detect the DNA double‐strand breaks, γH2AX foci‐positive cells per crypt was counted. A total of 30 crypts were examined per slide. The quantitative analysis of CD31 percent area positivity was calculated by ImageJ 1.54 g software (NIH, Bethesda, MD).

### 5.5 Determination of Villous Length and Crypt Depth

Quantitative analysis of villous height and crypt depth was performed in an unbiased, double‐blinded manner using coded digital images obtained from H&E‐stained intestinal sections. Measurements were conducted using ImageJ software (version 1.37). Crypt depth was assessed by measuring the distance, in pixels, from the crypt base to the crypt‐villus junction, while villous height was measured from the crypt‐villus junction to the tip of the villus. Pixel‐based measurements were subsequently converted to micrometers using a calibration factor of 1.46 pixels per µm.

### 5.6 Immunofluorescence Staining

Immunofluorescence staining was performed in paraffin‐embedded sections of mouse jejunum. Paraffin‐embedded sections were first deparaffinized and rehydrated through a graded series of alcohols. Before immunostaining, antigen retrieval was performed by heating slides in pH 6.0 citrate buffer at sub‐boiling temperature for 10 min in an 1x antigen retrieval solution (Dako, Cat No. S1699). Non‐specific antibody binding was blocked for 30 min by incubation with 5% normal goat serum in PBS (Invitrogen, Cat No. 50062Z). Tissue was stained using the following primary antibodies; anti‐ZO‐1 (Invitrogen, Cat No. 33–9100, 1:250 dilution), anti‐Claudin‐1 (Invitrogen, Cat No. 37–4900, 1:250 dilution), anti‐Muc‐2 (Santacruz, Cat No. sc‐15334, 1:200 dilution), anti‐Total OXPHOS (Abcam, Cat No. ab317270, 1:500 dilution) which targets key five complexes of the electron transport chain often known as complexes *I*–*V* involved in oxidative phosphorylation (OXPHOS), anti‐Olfm4 (Cell Signaling, Cat No. 39141, 1:250 dilution), diluted in 1% of blocking buffer and incubated overnight at 4°C in a dark humidified chamber. Sections were washed with 0.1% TBST and incubated with secondary antibody, such as donkey anti‐Rabbit IgG conjugated with Alexa Fluor 647 (Invitrogen, Cat. No. A32795), Goat anti‐rabbit IgG conjugated with Alexa Fluor 594 (Invitrogen, Cat. No. R37117), Goat anti‐Mouse IgG conjugated with Alexa Fluor 594 (Invitrogen, Cat. No. A‐11005), 1:1000 diluted in 1% of blocking buffer and incubated in dark humidified chamber for 1 h. After being washed with 0.1% TBST, the sections were mounted in Vectashield Dapi‐containing mounting medium (Vector Labs., Cat. No. H1200) and Fluorescence images were acquired using a confocal microscope (Nikon A1RMP). Quantitative analysis of Zo‐1, Claudin‐1, Muc‐2, Total OXPHOS was performed by measuring the mean fluorescence intensity, while Olfm4 was quantified by counting Olfm4+ crypt cells per field (a minimum of 10 fields was counted) of the acquired images using ImageJ 1.54 g software (NIH, Bethesda, MD).

### 5.7 TUNEL Assay

Apoptotic cells were detected in situ using Terminal deoxynucleotidyl transferase‐mediated dUTP nick end labeling (TUNEL) staining. TUNEL staining was performed on paraffin‐embedded tissue sections according to the manufacturer's instructions using a TUNEL assay kit (Invitrogen, Cat. No. C10618). Following staining, sections were counterstained with DAPI and mounted using ProLong Gold Antifade mounting medium. Images were acquired using an Evident VS200 slide scanner (0.325 µm/pixel). For quantitative analysis, a total of 40 crypts were analyzed per slide.

### 5.8 Identification and Quantification of Lgr5^+^ Intestinal Stem Cells

To examine the survival of Lgr5+ crypt base columnar cells, Lgr5/eGFP‐IRES‐Cre‐ERT2; R26‐ACTB‐tdTomato‐EGFP mice were irradiated with ABI 21 Gy of Proton FLASH (dose‐rate 57.14–64.29 Gy/s) and Proton CONV (dose‐rate: 0.5–1.0 Gy/s) irradiation. 96 h of post‐irradiation jejunal tissue were collected and processed for cryo‐sectioning. To assess intestinal stem cell (ISC) survival, Lgr5^+^ crypt cells were identified based on enhanced green fluorescent protein (eGFP) expression, while overall crypt morphology and epithelial structure were visualized using tdTomato fluorescence expressed in all epithelial cell membranes. Quantification of Lgr5^+^ ISCs were performed by counting the number of eGFP^+^ crypts per field in a cross‐section. For each mouse, a minimum of 10 randomly selected fields were analyzed.

### 5.9 Mucin Staining by Alcian Blue

Alcian blue staining of deparaffinized and rehydrated mouse intestinal tissue was performed using the Alcian Blue Stain Kit (pH 2.5, Mucin Stain) (Abcam; Cat No. ab150662) according to the manufacturer's protocol and examined microscopically. Following radiation, Alcian blue staining revealed a marked reduction in goblet cells and mucin production, indicating disruption of the protective mucus layer, impaired epithelial differentiation, and compromised barrier function, hallmarks of radiation‐induced gastrointestinal syndrome [73]. Goblet cells positive for Alcian blue were quantified manually using the ImageJ 1.54 g point tool (NIH, Bethesda, MD), with the average number of goblet cells counted in ten randomly selected fields per slide for comparative analysis.

### 5.10 Detection of Reactive Oxygen Species

Superoxide‐associated reactive oxygen species (ROS) levels in irradiated intestinal tissue were assessed by dihydroethidium (DHE) (Invitrogen, Cat. No. D11347) fluorescence staining. DHE penetrates cell membranes and oxidizes to 2‐hydroxyethidium (emitting red fluorescence), which intercalates with DNA, serving as a key marker for oxidative stress in cells. Paraffin‐embedded intestinal sections from Proton FLASH or Proton CONV irradiated samples were first deparaffinized and rehydrated through a graded series of alcohols, followed by washing in PBS. The sections were then incubated with 10 µM DHE solution in a dark, humidified chamber at 37°C for 15 min. After incubation, slides were rinsed thoroughly with distill water to remove unbound DHE and mounted using Vectashield Dapi‐containing mounting medium (Vector Labs., Cat. No. H1200). Fluorescence images were acquired using a confocal microscope (Nikon A1RMP). Quantitative analysis of ROS levels was performed by measuring the mean fluorescence intensity of the acquired images using ImageJ 1.54 g software (NIH, Bethesda, MD).

### 5.11 Mouse Intestinal Crypt Isolation and Single‐Cell Suspension

Mouse Intestinal tissue was isolated and washed three times with cold calcium‐ and magnesium‐free Hank's Balanced Salt Solution (HBSS) (Gibco, Cat No. 14170161). The cleaned tissue was open longitudinally and washed again five times with cold HBSS. After clearing all the intestinal contents, tissue was cut into small fragments (approximately 3–5 mm in length) and incubated in 30 mM EDTA prepared in HBSS for 20 min on ice. Following incubation, the EDTA solution was discarded, and the tissue fragments were washed again in HBSS. To promote the release of epithelial cells, vigorous shaking was performed (2–3 shakes per second for 3–5 min), followed by suspension was filtered through a 70 µm cell strainer. The resulting crypt‐enriched fraction was centrifuged at 300 x g for 5 min at 4°C. Pelleted crypts were then enzymatically dissociated in 10 mL of Gentle dissociation buffer (StemCell technology) with gently shaking at rotatory shaker for 10 min at room temperature. Gentle pipetting was used to facilitate single‐cell dissociation, and transfer the dissociated cells were mixed with 20 mL of ice‐cold wash media containing DMEM/F12 media with 10 µM of ROCK inhibitor and filtered using 40 µm cell strainer. The resulting cell suspension was centrifugation at 4°C, 500 x g for 5 min and pelleted cells were resuspended in PBS with 2% BSA. Finally, cells were counted for the viability check.

### 5.12 Single‐Cell RNA Sequencing

To characterize the epithelial cell populations at single‐cell resolution to gain deeper insight into the regenerative and differentiation potential of the intestinal epithelium following 21 Gy of Proton FLASH (dose‐rate 57.14–64.29 Gy/s) and Proton CONV (dose‐rate: 0.5–1.0 Gy/s) irradiation, Single‐cell RNA sequencing was performed. Mouse intestine was isolated 72 h post‐irradiation and single cell suspension was prepared as described above. The 10X Genomics Single Cell 3’ Expression v3.1 library preparation was performed using the 10X Genomics Chromium X. The single cells suspension was validated for viability and cell concentration using the Countess II FL Automated Cell Counter (Life Technologies) targeting ≥75% cell viability. The cell suspension was filtered through a FLOWMI Cell Strainer, 40 µm (Thermofisher 50‐136‐7555) to yield a homogenous single cell suspension and adjusted to ∼1000 cells/µL to prepare cells for emulsification. The cell emulsification is performed with the 10X Chromium X using the Chromium Next GEM Single Cell 3’ GEM Library & Gel Bead Kit v3.1 (10X Genomics 1000120) and Chromium Next GEM Chip G Single Cell Kit (10X Genomics 1000127). The RT reaction to generate 10X barcoded single‐stranded cDNA, in the single cell containing GEMs, is performed using an Eppendorf MasterCycler Pro thermal cycler. The QC and quantification of the cDNA is determined using the Agilent TapeStation 4200 HS D5000 ScreenTape assay (Agilent 5067–5592).

3′ gene expression libraries were prepared using 10x Genomics protocols. Double‐stranded cDNA was fragmented, end‐repaired, A‐tailed, and ligated with Illumina‐compatible Unique Dual Index (UDI) adapters (Dual Index Kit TT Set A, 10X Genomics, 1000215), followed by indexing PCR. Libraries were validated on the Agilent TapeStation 4200 (D1000 ScreenTape, 5067–5582) and quantified using Roche LightCycler 96 with FastStart Essential Green Master Mix and KAPA Library Quantification Kit (KAPA, KK4903). Libraries were normalized to 2 nM, pooled, and sequenced on an Illumina NovaSeq X Plus using a 100‐cycle kit (20104703) with a custom run format (Read1: 28 cycles, i7: 10 cycles, i5: 10 cycles, Read2: 90 cycles). Base calling, demultiplexing, and gene expression analysis were performed using Cell Ranger and Loupe Browser (10x Genomics).

### 5.13 Single‐Cell RNA Sequencing Data Processing and Analysis

Single‐cell RNA sequencing libraries were processed by aligning raw sequencing reads to the mm10 (mouse) reference genome and quantifying gene expression using Cell Ranger (v.7.1.0) with default parameters. Downstream analysis was performed on filtered features counts generated by Cell Ranger. Low‐quality cells were removed using the following thresholds: number of genes per cell ≤ 250 or ≥ 9000, log10(Genes/UMI) < 0.8, or percent mitochondrial reads per cell > 10%. A gene was removed if it was not detected in at least 10 cells. Seurat (v.5.0.1) [74] was used to normalize samples with the log‐normalization method and integrated using Harmony. The integrated dataset was then clustered using the Louvain algorithm and a resolution of 1. First, we identified epithelial cell clusters using *Epcam*. The epithelial cells were extracted to create a dataset. For dataset, we integrated and clustered the cells to further identify epithelial cell populations. A resolution of 1 was used to cluster the epithelial cells. Epithelial cell clusters were annotated based on the expression of known markers as previously described [27]. Crypt base columnar cells (CBC) were identified by the expression of stem cell genes Lgr5, Smoc2, Ascl2, and revival stem cells (revSC) through the expression of Ly6a, Clu, Areg, and Anxa. Transit amplifying (TA) cells had high expressions of Top2a, Mif, Mki67, and Pcna. Dclk1 and Kctd12 identified Tuft cells, while Chga and Chgb identified enteroendocrine (EE) cells. Goblet cells were identified with Muc2, Fcgbp, Guca2a, and Agr2. Paneth cells were identified by Defa22. Enterocyte clusters were identified through expression of Fabp1, Krt20, and Alpi (Figure S10C). The cell cycle phase was predicted for each cell using the CellCycleScoring function in Seurat. Differential gene expression (DGE) analysis was conducted for specific cell populations comparing the expression within a population between experimental groups (e.g., CBC cells: non‐irradiated vs. FLASH‐irradiated). A Wilcoxon rank sum test was used to test for DGE, and a gene was considered significantly different if the adjusted *p*‐value < 0.05, log2FC > 0.5 and proportion of cells expressing the gene > 0.1. Pathway over‐representation analysis was conducted using the clusterProfiler package in R with the Reactome and KEGG pathways and MSigDb Hallmark gene sets. A pathway was considered significant if the *p*‐value < 0.05. To predict the differentiation state of each cell, we used CytoTRACE [35], which outputs a stemness score ranging from 0 to 1. To have comparable stemness scores across all samples, we used the iCytoTRACE function in R.

### 5.14 Metagenomic Analysis

The mouse metagenomic analysis was performed on the mouse irradiated using ABI 18 Gy with Proton FLASH (dose‐rate: 57.14–64.29 and 43.21–49.38 Gy/s) and Proton CONV (dose‐rate: 0.5–1.0 Gy/s) irradiation. Fecal samples were collected at 24 and 72 h in a sterile microcentrifuge tube and immediately snap freeze in liquid nitrogen. After that samples were kept at ‐80°C until sent for metagenomic analysis at CosmosID, Inc. (Germantown, MD).

### 5.15 DNA Extraction, Library Preparation, and Sequencing

DNA from all the fecal samples were isolated using the QIAGEN DNeasy PowerSoil Pro Kit, according to the manufacturer's protocol. Isolated DNA was quantified using Qubit Flex fluorometer and Qubit dsDNA Assay Kit (Thermofisher Scientific). DNA libraries were prepared using the Watchmaker DNA Library Prep Kit (7K0019‐1K). Genomic DNA was fragmented using a master mix of Watchmaker Frag/AT Buffer and Frag/AT Enzyme

Mix. IDT xGen UDI Primers and IDT Stubby Adapters were added to each sample followed by 7 cycles of PCR to construct the DNA libraries. The final DNA libraries were purified using CleanNGS magnetic beads (CleanNA) and eluted in nuclease‐free water. Following elution, the libraries were quantified using the Qubit fluorometer dsDNA HS Assay Kit. Libraries were then sequenced on an Illumina NovaSeq X platform at 2×150 bp.

### 5.16 Metagenomic Taxonomic Profiling Using Kepler

Taxonomic profiling was performed using the patented Kepler multi‐kingdom algorithm, which integrates three core components: (1) a pre‐computed biomarker database (GenBook), (2) k‐mer‐based taxonomic classification, and (3) probabilistic abundance estimation. In the database construction phase, high‐quality microbial genomes across Archaea, Bacteria, Fungi, Protists, and Viruses were curated based on completeness, contamination, and assembly quality. Genomes were filtered to remove low‐complexity regions, prophages, plasmids, and host contaminants. Genomic content was split into variable‐length n‐mers and structured phylogenetically, with shared and unique biomarkers mapped across a tree‐like hierarchy. For classification, sample reads were decomposed into k‐mers and matched against reference biomarkers from GenBook, rapidly narrowing the candidate taxa list by excluding non‐matching genomes. This enabled high‐resolution taxonomic assignments down to sub‐species level (ANI < 0.003). A refined abundance estimation was performed using a probabilistic Smith–Waterman algorithm on candidate taxa, allowing resolution of ambiguous reads through weighted assignment and iterative maximum likelihood estimation. This two‐tiered approach ensured accurate, low‐variance abundance estimates and high classification precision across microbial kingdoms.

### 5.17 Functional Profiling of Metagenomic Reads

Metagenomic reads were quality‐filtered and adapter‐trimmed using BBduk [75]. High‐quality reads were translated and aligned to the UniRef90 database (UniProt) [76], which clusters non‐redundant protein sequences at ≥90% identity and ≥80% alignment coverage. Functional assignments were weighted by mapping quality, gene coverage, and sequence length to estimate community‐wide gene family abundances, following the approach of Franzosa et al. (2018) [77]. Gene families were annotated to MetaCyc [78] reactions for reconstruction and quantification of metabolic pathways. Additional annotations included Enzyme Commission (EC) numbers, Pfam domains, CAZy enzymes, and Gene Ontology (GO) terms to provide a broad functional overview. Abundance values were normalized across samples using Total‐Sum Scaling (TSS) to obtain “copies per million” (CPM), facilitating cross‐sample comparisons.

To assess potential cage effects on gut microbial population, β‐diversity analysis was performed on fecal samples from non‐irradiated control mice grouped by cage. Bray–Curtis PCoA followed by PERMANOVA demonstrated no significant cage‐associated clustering (PERMANOVA, *p* = 0.393), indicating that housing conditions did not significantly influence gut microbial composition in non‐irradiated control mice (Figure S16).

### 5.18 Fecal Microbiota Transplant (FMT) Study

The mouse fecal microbiota transplant study was performed on the recipient mouse irradiated using ABI 18 Gy with Proton CONV (dose‐rate: 0.5–1.0 Gy/s) irradiation. Fecal samples were collected in a sterile microcentrifuge tube from donor mice either non‐irradiated and/or irradiated with 18 Gy of Proton FLASH (dose‐rate 57.14–64.29 Gy/s) and/or Proton CONV (dose‐rate: 0.5–1.0 Gy/s) irradiation. After collecting, fecal samples were snap frozen immediately in liquid nitrogen and stored at ‐80°C until FMT study was performed. Fecal pellets from donor mice were thawed and processed in sterile PBS (2 pellets/mL of PBS) under sterile conditions by vortexing followed by centrifugation at 500 g for 5 min to remove the debris. The supernatant was than collected in a fresh sterile tube for oral administration in the mice (100 µL/ mouse).

Mice receiving FMT on day 1 and 3 post‐irradiations (D1, D3) were euthanized on day 4 to collect the GI tissue for histological processing. For survival study, animals were subjected to FMT on day 1, 3, and 5 post‐irradiations (D1, D3, and D5). All the mice were monitored daily for activity levels and survival analysis was performed until day 30 following irradiation.

### 5.19 Gut Permeability Marker Assessment

To analyze the gut epithelial barrier disruption, the mouse plasma levels of circulating LPS‐binding protein (LBP) and sCD14 (CD14) were analyzed as gut permeability marker. The Mouse LPS‐binding protein and sCD14 levels were measured in plasma samples using commercially available mouse ELISA kits for LPS‐binding protein (Abcam, Cat No. ab269542) and sCD14 (R&D Systems; Cat No. MC140) as per the manufacturer instructions. The average duplicate readings were calculated for standard, and blank, while each sample was analyzed in three biological replicates.

### 5.20 Quantitative Real‐Time PCR

qPCR was performed to determine the oxidative stress gene and Bmi gene expression in intestinal epithelial cells following ABI of 21 Gy of Proton FLASH (dose‐rate 57.14–64.29 Gy/s) and Proton CONV (dose‐rate: 0.5–1.0 Gy/s) irradiation. After isolation of epithelial cells as described above, total RNA was isolated from all the samples using RNeasy micro/mini kit from (Qiagen, Germantown, MD). The concentration and purity of the extracted RNA were checked using a NanoDrop spectrometer (Thermo Scientific, Waltham, MA). 1 µg of total RNA was reverse transcribed using RNA to cDNA EcoDry Premix (Double Primed) (Takara Bio USA Inc., San Jose, CA). The reaction mixture was incubated for 1 h at 42°C; incubation was stopped at 70°C for 10 min. Quantitative real‐time PCR (qPCR) was performed using the QuantStudio 7 Flex Real Time PCR System (Applied Biosystems, New York, NY) and SYBR Green Supermix (Bio‐Rad, Hercules, CA) with specific primers to the target genes in a 20 µL final reaction volume. GAPDH was used as a reference gene for sample normalization. The delta‐delta threshold cycle (ΔΔCt) method was used to calculate the fold change expression in mRNA level in the samples. All the primer sequences used in this study are listed in Table S1.

### 5.21 Statistical Analysis

Mice survival/mortality was analyzed by Kaplan–Meier Log‐rank (Mantel–Cox) test. For intestinal sampling, regions were chosen at random for digital acquisition for quantitation. All quantitative data were analyzed using GraphPad Prism (v10.4.2). Comparisons between groups were made using unpaired two‐tailed Student's *t*‐test. A *p‐*value <0.05 was considered statistically significant. Data are presented as mean ± SEM. For scRNA‐seq analysis, to test for differences in cell populations between non‐irradiated (Control), Proton FLASH, and Proton CONV irradiation, we performed a Dirichlet‐multinomial regression model since the cell proportions are compositional data. The models were fitted in R using the DirichletReg package with the alternative parameterization. To account for testing multiple comparisons, adjusted *p*‐values using the Benjamini–Hochberg procedure. A cell population was considered significantly different between two conditions if the adjusted *p*‐value < 0.05.

## Author Contributions

S.S. conceived the project and designed the research. R.M.C. and P.B. performed the experiments and interpreted the results. J.S., Y.L., and K.G. performed the radiation, R.M.C., P.B., S.R., E.S., and K.L.C. analyzed the data, R.M.C. and S.S. wrote the first draft of the manuscript. R.M.C., P.B. E.S., K.L.C, and S.S. edited the manuscript. S.K., D.K., and R.C. provided the inputs. S.S. provided essential reagents, tools, and funding. All authors approved the manuscript.

## Funding

This work was supported by a sponsored Research Agreement from IBA to perform preclinical research on FLASH Proton Radiotherapy (KUMC‐IBA collaborative fund) and KUCC CCSG (P30 CA168524).

## Conflicts of Interest

The authors declare no conflicts of interest.

## Supporting information




**Supporting File**: advs74873‐sup‐0001‐SuppMat.docx.

## Data Availability

The data that support the findings of this study are available from the corresponding author upon reasonable request.

## References

[1] A. K. Shadad , F. J. Sullivan , J. D. Martin , and L. J. Egan , “Gastrointestinal Radiation Injury: Symptoms, Risk Factors and Mechanisms,” World Journal of Gastroenterology 19, no. 2 (2013): 185–198, 10.3748/wjg.v19.i2.185. PMC3547560 23345941

[2] M. Hauer‐Jensen , J. W. Denham , and H. J. Andreyev , “Radiation Enteropathy—Pathogenesis, Treatment and Prevention,” Nature Reviews Gastroenterology & Hepatology 11, no. 8 (2014): 470–479, 10.1038/nrgastro.2014.46. PMC4346191 24686268

[3] R. M. Chugh , P. Bhanja , R. Zitter , S. Gunewardena , R. Badkul , and S. Saha , “Modulation of β‐Catenin Promotes WNT Expression in Macrophages and Mitigates Intestinal Injury,” Cell Communication and Signaling 23, no. 1 (2025): 78, 10.1186/s12964-025-02065-7. PMC11818365 39934819

[4] R. C. Zitter , R. M. Chugh , P. Bhanja , B. F. Kimler , and S. Saha , “LGR5+ Intestinal Stem Cells Display Sex‐Dependent Radiosensitivity,” Cells 13, no. 1 (2023): 46, 10.3390/cells13010046. PMC10778194 38201250

[5] E. J. Hall and D. J. Brenner , “The Dose‐Rate Effect Revisited: Radiobiological Considerations of Importance in Radiotherapy,” International Journal of Radiation Oncology*Biology*Physics 21, no. 6 (1991): 1403–1414, 10.1016/0360-3016(91)90314-T. 1938548

[6] P. Montay‐Gruel , M. M. Acharya , P. Gonçalves Jorge , et al., “Hypofractionated FLASH‐RT as An Effective Treatment Against Glioblastoma That Reduces Neurocognitive Side Effects in Mice,” Clinical Cancer Research 27, no. 3 (2021): 775–784, 10.1158/1078-0432.CCR-20-0894. PMC7854480 33060122

[7] K. Levy , S. Natarajan , J. Wang , et al., “Abdominal FLASH Irradiation Reduces Radiation‐Induced Gastrointestinal Toxicity for the Treatment of Ovarian Cancer in Mice,” Scientific Reports 10, no. 1 (2020): 21600, 10.1038/s41598-020-78017-7. PMC7728763 33303827

[8] J. Ma , H. Gao , X. Shen , X. Bai , and M. Tang , “A FLASH Model of Radiolytic Oxygen Depletion and Reactive Oxygen Species for Differential Tumor and Normal‐Tissue Response,” medRxiv (2023): 23297337, 10.1101/2023.10.20.23297337.

[9] A. Hu , R. Qiu , W. B. Li , et al., “Radical Recombination and Antioxidants: A Hypothesis on the FLASH Effect Mechanism,” International Journal of Radiation Biology 99, no. 4 (2023): 620–628, 10.1080/09553002.2022.2110307. 35938944

[10] S. Cui and G. Pratx , “3D Computational Model of Oxygen Depletion Kinetics in Brain Vasculature During FLASH RT and Its Implications for In Vivo Oximetry Experiments,” Medical Physics 49, no. 6 (2022): 3914–3925, 10.1002/mp.15642. 35393643

[11] E. D. Bankaitis , A. Ha , C. J. Kuo , and S. T. Magness , “Reserve Stem Cells in Intestinal Homeostasis and Injury,” Gastroenterology 155, no. 5 (2018): 1348–1361, 10.1053/j.gastro.2018.08.016. PMC7493459 30118745

[12] D. Hu , H. Yan , X. C. He , and L. Li , “Recent Advances in Understanding Intestinal Stem Cell Regulation,” F1000Research 8 (2019): 72, 10.12688/f1000research.16793.1. PMC6343229 30705753

[13] T. L. Lim , C. Morral , I. I. Verginadis , et al., “Early Inflammation and Interferon Signaling Direct Enhanced Intestinal Crypt Regeneration after Proton FLASH Radiotherapy,” bioRxiv (2024),608284, 10.1101/2024.08.16.608284.

[14] D. J. Sanders , S. Inniss , G. Sebepos‐Rogers , F. Z. Rahman , and A. M. Smith , “The Role of the Microbiome in Gastrointestinal Inflammation,” Bioscience Reports 41, no. 6 (2021), 10.1042/BSR20203850. PMC8201460 34076695

[15] Y. Jian , D. Zhang , M. Liu , Y. Wang , and Z. X. Xu , “The Impact of Gut Microbiota on Radiation‐Induced Enteritis,” Frontiers in Cellular and Infection Microbiology 11 (2021): 586392, 10.3389/fcimb.2021.586392. PMC8358303 34395308

[16] D. Mitra , G. K. Armijo , E. H. Ober , S. M. Baker , H. C. Turner , and C. G. Broustas , “MIIST305 Mitigates Gastrointestinal Acute Radiation Syndrome Injury and Ameliorates Radiation‐Induced Gut Microbiome Dysbiosis,” Gut Microbes 17, no. 1 (2025): 2458189, 10.1080/19490976.2025.2458189. PMC11817531 39930324

[17] F. Paris , Z. Fuks , A. Kang , et al., “Endothelial Apoptosis as the Primary Lesion Initiating Intestinal Radiation Damage in Mice,” Science 293, no. 5528 (2001): 293–297, 10.1126/science.1060191. 11452123

[18] D. Dahlgren and H. Lennernäs , “Review on the Effect of Chemotherapy on the Intestinal Barrier: Epithelial Permeability, Mucus and Bacterial Translocation,” Biomedicine & Pharmacotherapy 162 (2023): 114644, 10.1016/j.biopha.2023.114644. 37018992

[19] V. J. Gonzalez‐Mercado , S. Marrero , J. Pérez‐Santiago , et al., “Association of Radiotherapy‐Related Intestinal Injury and Cancer‐Related Fatigue: A Brief Review and Commentary,” Puerto Rico Health Sciences Journal 40, no. 1 (2021): 6–11. PMC9109698 33876912

[20] R. R. Schumann , S. R. Leong , G. W. Flaggs , et al., “Structure and Function of Lipopolysaccharide Binding Protein,” Science 249, no. 4975 (1990): 1429–1431, 10.1126/science.2402637. 2402637

[21] M. Gaber , A. S. Wilson , A. E. Millen , et al., “Visceral Adiposity in Postmenopausal Women Is Associated With a Pro‐Inflammatory Gut Microbiome and Immunogenic Metabolic Endotoxemia,” Microbiome 12, no. 1 (2024): 192, 10.1186/s40168-024-01901-1. PMC11453046 39367431

[22] S. Saha , E. Aranda , Y. Hayakawa , et al., “Macrophage‐Derived Extracellular Vesicle‐Packaged WNTs Rescue Intestinal Stem Cells and Enhance Survival After Radiation Injury,” Nature Communications 7 (2016): 13096, 10.1038/ncomms13096. PMC5065628 27734833

[23] P. Bhanja , A. Norris , P. Gupta‐Saraf , A. Hoover , and S. Saha , “BCN057 induces Intestinal Stem Cell Repair and Mitigates Radiation‐Induced Intestinal Injury,” Stem Cell Research & Therapy 9, no. 1 (2018): 26, 10.1186/s13287-017-0763-3. PMC5797353 29394953

[24] S. Saha , P. Bhanja , R. Kabarriti , L. Liu , and A. A. Alfieri , “Bone Marrow Stromal Cell Transplantation Mitigates Radiation‐Induced Gastrointestinal Syndrome in Mice,” PLoS ONE 6, no. 9 (2011): 24072, 10.1371/journal.pone.0024072. PMC3174150 21935373

[25] N. Barker , M. van de Wetering , and H. Clevers , “The Intestinal Stem Cell,” Genes & Development 22, no. 14 (2008): 1856–1864, 10.1101/gad.1674008. PMC2735277 18628392

[26] A. Ayyaz , S. Kumar , B. Sangiorgi , et al., “Single‐Cell Transcriptomes of the Regenerating Intestine Reveal a Revival Stem Cell,” Nature 569, no. 7754 (2019): 121–125, 10.1038/s41586-019-1154-y. 31019301

[27] C. Morral , A. Ayyaz , H. Kuo , et al., “p53 Promotes Revival Stem Cells in the Regenerating Intestine After Severe Radiation Injury,” Nature Communications 15, no. 1 (2024): 3018, 10.1038/s41467-024-47124-8. PMC11001929 38589357

[28] A. Gregorieff , D. Pinto , H. Begthel , O. Destrée , M. Kielman , and H. Clevers , “Expression Pattern of Wnt Signaling Components in the Adult Intestine,” Gastroenterology 129, no. 2 (2005): 626–638, 10.1016/j.gastro.2005.06.007. 16083717

[29] D. L. Daniels and W. I. Weis , “β‐Catenin Ddirectly Displaces Groucho/TLE Repressors From Tcf/Lef in Wnt‐Mediated Transcription Activation,” Nature Structural & Molecular Biology 12, no. 4 (2005): 364–371, 10.1038/nsmb912. 15768032

[30] H. Brantjes , J. Roose , M. van De Wetering , and H. Clevers , “All Tcf HMG Box Transcription Factors Interact With Groucho‐Related Co‐Repressors,” Nucleic Acids Research 29, no. 7 (2001): 1410–1419, 10.1093/nar/29.7.1410. PMC31284 11266540

[31] W. H. Lien , L. Polak , M. Lin , K. Lay , D. Zheng , and E. Fuchs , “In Vivo Transcriptional Governance of Hair Follicle Stem Cells by Canonical Wnt Regulators,” Nature Cell Biology 16, no. 2 (2014): 179–190, 10.1038/ncb2903. PMC3984009 24463605

[32] Y. Zhang , C. Liu , X. Duan , et al., “CREPT/RPRD1B, A Recently Identified Novel Protein Highly Expressed in Tumors, Enhances the β‐Catenin·TCF4 Transcriptional Activity in Response to Wnt Signaling,” Journal of Biological Chemistry 289, no. 33 (2014): 22589–22599, 10.1074/jbc.M114.560979. PMC4132767 24982424

[33] L. Yang , H. Yang , Y. Chu , et al., “CREPT Is Required for Murine Stem Cell Maintenance During Intestinal Regeneration,” Nature Communications 12, no. 1 (2021): 270, 10.1038/s41467-020-20636-9. PMC7801528 33431892

[34] T. Lan , M. Li , X. Duan , et al., “Regulation of Revival Stem Cell Differentiation by CREPT/RPRD1B During Intestinal Regeneration,” Cell & Bioscience 15, no. 1 (2025): 98, 10.1186/s13578-025-01434-6. PMC12232850 40624542

[35] G. S. Gulati , S. S. Sikandar , D. J. Wesche , et al., “Single‐Cell Transcriptional Diversity Is a Hallmark of Developmental Potential,” Science 367, no. 6476 (2020): 405–411, 10.1126/science.aax0249. PMC7694873 31974247

[36] M. Fink and J. L. Wrana , “Regulation of Homeostasis and Regeneration in the Adult Intestinal Epithelium by the TGF‐β Superfamily,” Developmental Dynamics 252, no. 4 (2023): 445–462, 10.1002/dvdy.500. 35611490

[37] Z. Xie , M. Zhang , G. Zhou , et al., “Emerging Roles of the Hedgehog Signalling Pathway in Inflammatory Bowel Disease,” Cell Death Discovery 7, no. 1 (2021): 314, 10.1038/s41420-021-00679-7. PMC8548344 34702800

[38] U. Herbig , J. W. Griffith , and E. Fanning , “Mutation of Cyclin/Cdk Phosphorylation Sites in HsCdc6 Disrupts a Late Step in Initiation of DNA Replication in Human Cells,” Molecular Biology of the Cell 11, no. 12 (2000): 4117–4130, 10.1091/mbc.11.12.4117. PMC15061 11102512

[39] L. Qin , M. Fan , D. Candas , et al., “CDK1 Enhances Mitochondrial Bioenergetics for Radiation‐Induced DNA Repair,” Cell Reports 13, no. 10 (2015): 2056–2063, 10.1016/j.celrep.2015.11.015. PMC4684969 26670043

[40] K. L. Edelblum , J. A. Goettel , T. Koyama , S. J. McElroy , F. Yan , and D. B. Polk , “TNFR1 Promotes Tumor Necrosis Factor‐Mediated Mouse Colon Epithelial Cell Survival Through RAF Activation of NF‐κB,” Journal of Biological Chemistry 283, no. 43 (2008): 29485–29494, 10.1074/jbc.M801269200. PMC2570867 18713739

[41] P. E. Dubé , S. Punit , and D. B. Polk , “Redeeming an Old Foe: Protective as Well as Pathophysiological Roles for Tumor Necrosis Factor in Inflammatory Bowel Disease,” American Journal of Physiology Gastrointestinal and Liver Physiology 308, no. 3 (2015): 61–70. 10.1152/ajpgi.00142.2014 PMC4312954 25477373

[42] S. Saini and P. Gurung , “A Comprehensive Review of Sensors of Radiation‐Induced Damage, Radiation‐Induced Proximal Events, and Cell Death,” Immunological Reviews 329, no. 1 (2025): 13409, 10.1111/imr.13409. PMC11742653 39425547

[43] V. Grilj , R. J. Leavitt , M. El Khatib , et al., “In Vivo Measurements of Change in Tissue Oxygen Level During Irradiation Reveal Novel Dose Rate Dependence,” Radiotherapy and Oncology 201 (2024): 110539. 10.1016/j.radonc.2024.110539 39299575

[44] V. Favaudon , R. Labarbe , and C. L. Limoli , “Model Studies of the Role of Oxygen in the FLASH Effect,” Medical Physics 49, no. 3 (2022): 2068–2081, 10.1002/mp.15129. PMC8854455 34407219

[45] J. C. L. Chow and H. E. Ruda , “Mechanisms of Action in FLASH Radiotherapy: A Comprehensive Review of Physicochemical and Biological Processes on Cancerous and Normal Cells,” Cells 13, no. 10 (2024): 835, 10.3390/cells13100835. PMC11120005 38786057

[46] H. K. Krishnamurthy , I. Rajavelu , M. Pereira , et al., “Inside the Genome: Understanding Genetic Influences on Oxidative Stress,” Frontiers in Genetics 15 (2024): 2024. 10.3389/fgene.2024.1397352 PMC11231378 38983269

[47] N. Bhandari , D. Acharya , A. Chatterjee , et al., “MRPL47 Deficiency Drives Mitochondrial Dysfunction via ROS/p38‐MAPK/CDKN1A Signaling in Non‐Small Cell Lung Cancer,” bioRxiv (2025), 10.1101/2025.02.17.638626. PMC12809090 41407038

[48] M. P. Murphy , H. Bayir , V. Belousov , et al., “Guidelines for Measuring Reactive Oxygen Species and Oxidative Damage in Cells and In Vivo,” Nature Metabolism 4, no. 6 (2022): 651–662, 10.1038/s42255-022-00591-z. PMC9711940 35760871

[49] U. S. Srinivas , B. W. Q. Tan , and B. A. Vellayappan , “ROS and the DNA Damage Response in Cancer,” Redox Biology 25 (2019): 101084, 10.1016/j.redox.2018.101084. PMC6859528 30612957

[50] D. Nag , P. Bhanja , R. Riha , et al., “Auranofin Protects Intestine Against Radiation Injury by Modulating p53/p21 Pathway and Radiosensitizes Human Colon Tumor,” Clinical Cancer Research 25, no. 15 (2019): 4791–4807, 10.1158/1078-0432.CCR-18-2751. PMC9159899 30940656

[51] E. Kang , J. Kim , Y. E. Kim , et al., “The Secreted Protein Amuc_1409 From Akkermansia Muciniphila Improves Gut Health Through Intestinal Stem Cell Regulation,” Nature Communications 15, no. 1 (2024): 2983, 10.1038/s41467-024-47275-8. PMC10998920 38582860

[52] S. Kim , Y. Shin , T. Kim , et al., “Mucin Degrader Akkermansia Muciniphila Accelerates Intestinal Stem Cell‐Mediated Epithelial Development,” Gut Microbes 13, no. 1 (2021): 1–20, 10.1080/19490976.2021.1892441. PMC7946046 33678130

[53] Y. Shin , S. Han , J. Kwon , et al., “Roles of Short‐Chain Fatty Acids in Inflammatory Bowel Disease,” Nutrients 15, no. 20 (2023): 4466, 10.3390/nu15204466. PMC10609902 37892541

[54] W. Fusco , M. B. Lorenzo , M. Cintoni , et al., “Short‐Chain Fatty‐Acid‐Producing Bacteria: Key Components of the Human Gut Microbiota,” Nutrients 15, no. 9 (2023): 2211, 10.3390/nu15092211. PMC10180739 37432351

[55] S. Ghosh , C. S. Whitley , B. Haribabu , and V. R. Jala , “Regulation of Intestinal Barrier Function by Microbial Metabolites,” Cellular and Molecular Gastroenterology and Hepatology 11, no. 5 (2021): 1463–1482. 10.1016/j.jcmgh.2021.02.007 PMC8025057 33610769

[56] A. Gutierrez , B. Pucket , and M. A. Engevik , “Bifidobacterium and the Intestinal Mucus Layer,” Microbiome Research Reports 2, no. 4 (2023): 36, 10.20517/mrr.2023.37. PMC10688832 38045921

[57] L. Wang , Y. Li , Y. J. Zhang , and L. H. Peng , “Intestinal Microecological Transplantation for a Patient With Chronic Radiation Enteritis: A Case Report,” World Journal of Gastroenterology 30, no. 19 (2024): 2603–2611, 10.3748/wjg.v30.i19.2603. PMC11135409 38817661

[58] W. Xiao , R. S. Wang , D. E. Handy , and J. Loscalzo , “NAD(H) and NADP(H) Redox Couples and Cellular Energy Metabolism,” Antioxidants & Redox Signaling 28, no. 3 (2018): 251–272, 10.1089/ars.2017.7216. PMC5737637 28648096

[59] I. R. Miousse , L. E. Ewing , C. M. Skinner , et al., “Methionine Dietary Supplementation Potentiates Ionizing Radiation‐Induced Gastrointestinal Syndrome,” American Journal of Physiology‐Gastrointestinal and Liver Physiology 318, no. 3 (2020): G439–G450, 10.1152/ajpgi.00351.2019. PMC7099489 31961718

[60] W. Wang , B. Cui , Y. Nie , L. Sun , and F. Zhang , “Radiation Injury and Gut Microbiota‐Based Treatment,” Protein & Cell 15, no. 2 (2024): 83–97, 10.1093/procel/pwad044. PMC10833463 37470727

[61] T. M. Yeung , L. A. Chia , C. M. Kosinski , and C. J. Kuo , “Regulation of Self‐Renewal and Differentiation by the Intestinal Stem Cell Niche,” Cellular and Molecular Life Sciences 68, no. 15 (2011): 2513–2523, 10.1007/s00018-011-0687-5. PMC4165857 21509540

[62] F. R. Tirado , P. Bhanja , E. Castro‐Nallar , X. D. Olea , C. Salamanca , and S. Saha , “Radiation‐Induced Toxicity in Rectal Epithelial Stem Cell Contributes to Acute Radiation Injury in Rectum,” Stem Cell Research & Therapy 12, no. 1 (2021): 63, 10.1186/s13287-020-02111-w. PMC7811242 33451351

[63] X. Cao , R. Zhang , T. V. Esipova , et al., “Quantification of Oxygen Depletion during FLASH Irradiation In Vitro and In Vivo,” International Journal of Radiation Oncology*Biology*Physics 111, no. 1 (2021): 240–248, 10.1016/j.ijrobp.2021.03.056. PMC8338745 33845146

[64] B. Ortmann , J. Druker , and S. Rocha , “Cell Cycle Progression in Response to Oxygen Levels,” Cellular and Molecular Life Sciences 71, no. 18 (2014): 3569–3582, 10.1007/s00018-014-1645-9. PMC4143607 24858415

[65] A. E. Rose , R. T. Fansler , and W. Zhu , “Commensal Resilience: Ancient Ecological Lessons for the Modern Microbiota,” Infection and Immunity 93, no. 6 (2025): 0050224, 10.1128/iai.00502-24. PMC12150765 40387449

[66] Y. Sun , X. Wang , L. Li , et al., “The Role of Gut Microbiota in Intestinal Disease: From an Oxidative Stress Perspective,” Frontiers in Microbiology 15 (2024): 1328324, 10.3389/fmicb.2024.1328324. PMC10899708 38419631

[67] Z. Lu and J. A. Imlay , “When Anaerobes Encounter Oxygen: Mechanisms of Oxygen Toxicity, Tolerance and Defence,” Nature Reviews Microbiology 19, no. 12 (2021): 774–785, 10.1038/s41579-021-00583-y. PMC9191689 34183820

[68] C. Mo , X. Lou , J. Xue , et al., “The Influence of Akkermansia Muciniphila on Intestinal Barrier Function,” Gut Pathogens 16, no. 1 (2024): 41, 10.1186/s13099-024-00635-7. PMC11297771 39097746

[69] X. Lin , M. Xu , R. Lan , et al., “Gut Commensal *Alistipes* Shahiiimproves Experimental Colitis in Mice With Reduced Intestinal Epithelial Damage and Cytokine Secretion,” mSystems 10, no. 3 (2025): e0160724, 10.1128/msystems.01607-24. PMC11915872 39936902

[70] L. Lu , F. Li , Y. Gao , S. Kang , J. Li , and J. Guo , “Microbiome in Radiotherapy: An Emerging Approach to Enhance Treatment Efficacy and Reduce Tissue Injury,” Molecular Medicine 30, no. 1 (2024): 105, 10.1186/s10020-024-00873-0. PMC11264922 39030525

[71] M. D. Muzumdar , B. Tasic , K. Miyamichi , L. Li , and L. Luo , “A Global Double‐Fluorescent Cre Reporter Mouse,” Genesis 45, no. 9 (2007): 593–605, 10.1002/dvg.20335. 17868096

[72] C. S. Potten , C. Booth , and D. M. Pritchard , “The Intestinal Epithelial Stem Cell: The Mucosal Governor,” International Journal of Experimental Pathology 78, no. 4 (1997): 219–243, 10.1046/j.1365-2613.1997.280362.x. PMC2694540 9505935

[73] X. Zhu , Y. Li , X. Tian , et al., “REGγ Mitigates Radiation‐Induced Enteritis by Preserving Mucin Secretion and Sustaining Microbiome Homeostasis,” The American Journal of Pathology 194, no. 6 (2024): 975–988, 10.1016/j.ajpath.2024.02.008. 38423356

[74] Y. Hao , T. Stuart , M. H. Kowalski , et al., “Dictionary Learning for Integrative, Multimodal and Scalable Single‐Cell Analysis,” Nature Biotechnology 42, no. 2 (2024): 293–304, 10.1038/s41587-023-01767-y. PMC10928517 37231261

[75] B. Bushnell , “BBDuk Guide—DOE Joint Genome Institute” Accessed August 1, (2021), https://jgi.doe.gov/data‐and‐tools/bbtools/bb‐tools‐user‐guide/bbduk‐guide/.

[76] The UniProt Consortium , “UniProt: The Universal Protein Knowledgebase,” Nucleic Acids Research 45 D1 (2016): D158–D169. 10.1093/nar/gkw1099 PMC5210571 27899622

[77] E. A. Franzosa , L. J. McIver , G. Rahnavard , et al., “Species‐Level Functional Profiling of Metagenomes and Metatranscriptomes,” Nature Methods 15, no. 11 (2018): 962–968, 10.1038/s41592-018-0176-y. PMC6235447 30377376

[78] R. Caspi , R. Billington , L. Ferrer , et al., “The MetaCyc Database of Metabolic Pathways and Enzymes and the BioCyc Collection of Pathway/Genome Databases,” Nucleic Acids Research 44, no. D1 (2016): D471–D480, 10.1093/nar/gkv1164. PMC4702838 26527732

