# Supplementary material for: Proton FLASH Exposure Preserves Gut Commensal Microbiomes and Spares Intestinal Stem Cells
Source: Adv Sci (Weinh). 2026 Mar 28;13(31):e19249. doi: 10.1002/advs.202519249 (PMC13587989; doi:10.1002/advs.202519249)
Supplement: Supplementary file 1 [file ADVS-13-e19249-s001.docx]

**Supplementary Figures:**

***Supplementary Figure 1:*** *Proton FLASH irradiation preserves intestinal morphology following abdominal irradiation (ABI). (A) Schematic representation of experimental design. Mice received 18 Gy ABI using either Proton CONV (dose rate: 0.5-1.0 Gy/s) or Proton FLASH (dose rate: 43.21-49.38 Gy/s) irradiation, followed by histological analysis of jejunal sections at 72 hours post-irradiation. (B) Representative H&E-stained jejunal cross-sections from Proton CONV- and Proton FLASH-irradiated mice shows improved mucosal preservation in Proton FLASH-irradiated group (C, D) Violin plot demonstrating quantification of villus length and crypt depth. Proton FLASH-irradiated tissues demonstrated significantly greater epithelial regeneration compared to Proton CONV irradiation. (B, E) showing the number of Ki67+ proliferative cells per crypt. Proton FLASH-irradiated mice demonstrate significantly higher Ki67+ cell counts compared to Proton CONV-irradiated animals. (F) The dose-response curve for Ki67+ cells with 18 and 21 Gy Proton FLASH (dose rate: 57.14-64.29 Gy/sec.) demonstrated Proton FLASH irradiation minimizes the radiation-induced decrease in crypt proliferation. Data presented as mean ± SD. (Significant; ****p < 0.00005). n=10 mice per group.*

 ***Supplementary Figure 2:*** *Quantification of radiation induced apoptosis in Crypt epithelial cells and determination of radiation effect on intestinal endothelial cells. (A) Confocal Microscopic images of mouse jejunal sections to determine Tunel positive crypt epithelial cells at 24 hours post-irradiation. TUNEL+ cells are Purple indicated with white arrow. Nuclei are stained with DAPI (stained blue). (B) Violin plot showing the number of TUNEL-positive cells per intestinal crypt (a total of 40 crypts were analyzed per slide) Proton CONV irradiation resulted in a significant increase in apoptotic cell death compared to non-irradiated controls, whereas Proton FLASH irradiation significantly reduced the number of TUNEL-positive cells relative to Proton CONV irradiation. (C) Light microscopic images of jejunal section subjected to Immunohistochemical staining of CD31 after 72 hours post-irradiation. D. Violin plot demonstrated the Proton CONV irradiation resulted in a significant decrease of CD31+ endothelial cells within the crypt/villous region compared to non-irradiated control. However, Proton FLASH irradiation spares the CD31+ endothelial cell population. Data presented as mean ± SD. (Significant; ****p < 0.00005, ***p < 0.0005). n=5 mice per group.*

 ***Supplementary Figure 3:*** *Proton FLASH irradiation preserves mucosal barrier function in mice. Serum level of circulating LPS-binding protein (LPS-BP) (A) and soluble CD14 (sCD14) (B) were assessed at 96 hours post-irradiation as markers of endotoxin-associated inflammatory signaling and epithelial barrier disruption. Bar graph demonstrated that Proton FLASH irradiation significantly reduced both LBP and CD14 levels in plasma compared to Proton CONV irradiation. (C) Light microscopic images of jejunal sections at 72 hours post-irradiation stained with Alcian blue to determine mucin-secreting goblet cells in the intestinal epithelium. (D) Violin plot demonstrated significant reduction in number of goblet cells with Proton CONV (dose rate: 0.5-1.0 Gy/s) irradiation (18-21 Gy) compared to Proton FLASH (dose rate: 57.14-64.29 Gy/sec.) irradiation. Data are presented as mean ± SD ( *p < 0.05, **p < 0.005, ****p < 0.00005). n= 5 mice per group.*

***Supplementary Figure 4:*** *Proton FLASH Irradiation preserves tight junction ZO-1 expression in the intestinal epithelium. (A) Confocal microscopic images of mouse jejunal sections to detect ZO-1 positive epithelial cells at 72 hours post-irradiation. ZO1 positive cells (stained purple in merged image) are indicated with arrow. Nuclei are stained (blue) with Dapi. (B) Violin plot demonstrated quantification of ZO-1 immunofluorescence intensity in intestinal epithelium with No IR, 18 Gy and 21 Gy of Proton FLASH (dose rate: 57.14-64.29 Gy/sec.) and Proton CONV (dose rate: 0.5-1.0 Gy/s) irradiation. Proton CONV irradiation with both the radiation dose 18 and 21 Gy significantly reduced ZO-1 expression compared with non-irradiated controls (No IR), whereas 18 and 21 Gy Proton FLASH irradiation significantly preserved ZO-1 levels compared to Proton CONV irradiation.* *ZO-1 positive signal was quantified by Image J and expressed as Mean Fluorescent Intensity (%). Data presented as mean ± SD. (Significant; ***p < 0.00005, **p<0.005).* *n= 5 mice per group.*

 ***Supplementary Figure 5:*** *Proton FLASH Irradiation maintains Claudin-1 expression in the intestinal epithelium. (A) Confocal microscopic images of mouse jejunal sections to detect Claudin-1 positive epithelial cells at 72 hours post-irradiation. Claudin-1 positive cells (stained purple in merged image) are indicated with arrow. Nuclei are stained (blue) with Dapi. (B) Violin plot demonstrated quantification of Claudin-1 immunofluorescence intensity in intestinal epithelium with No IR, 18 Gy and 21 Gy of Proton FLASH (dose rate: 57.14-64.29 Gy/sec.) and Proton CONV (dose rate: 0.5-1.0 Gy/s) irradiation. Proton CONV irradiation with both the radiation dose 18 and 21 Gy significantly reduced Claudin-1 expression compared with non-irradiated controls (No IR), whereas 18 and 21 Gy Proton FLASH irradiation significantly improved Claudin-1 levels compared to Proton CONV irradiation. Claudin-1 positive signal was quantified by Image J and expressed as Mean Fluorescent Intensity (%). Data presented as mean ± SD. (Significant; *p<0.05, **p<0.005, ***p < 0.0005). n= 5 mice per group.*

 ***Supplementary Figure 6:*** *Proton FLASH Irradiation preserves Muc-2 expression in the intestinal epithelium. (A) Confocal microscopic images of mouse jejunal sections to detect Muc-2 epithelial cells at 72 hours post-irradiation. Muc-2 positive cells (stained purple in merged image) are indicated with arrow. Nuclei are stained (blue) with Dapi. (B) Violin plot demonstrated quantification of Muc-2 immunofluorescence intensity in intestinal epithelium with No IR, 18 Gy and 21 Gy of Proton FLASH (dose rate: 57.14-64.29 Gy/sec.) and Proton CONV (dose rate: 0.5-1.0 Gy/s) irradiation. CONV irradiation with both the radiation dose 18 and 21 Gy significantly reduced Muc-2 expression compared with non-irradiated controls (No IR), whereas Proton FLASH irradiation significantly preserved Muc-2 levels compared to Proton CONV irradiation. Muc2 positive signal was quantified by Image J and expressed as Mean Fluorescent Intensity (%). Data presented as mean ± SD. (Significant; ***p < 0.0005). n= 5 mice per group.*

***Supplementary Figure 7:*** *Proton FLASH irradiation preserves intestinal stem cells. qPCR analysis of intestinal epithelial total RNA demonstrated higher expression of Bmi in mice exposed to Proton FLASH irradiated intestinal epithelial cells compared to Proton CONV irradiated cells. Data presented as mean ± SD. (Significant; *p < 0.05).*

 ***Supplementary Figure 8:*** *Proton FLASH irradiation preserves intestinal stem cells. (A) Schematic representation of experimental design using Lgr5-EGFP-CreERT2; R26-ACTB-tdTomato-EGFP reporter mice exposed to 21 Gy ABI using Proton FLASH (dose rate: 57.14-64.29 Gy/sec.) or Proton CONV (dose rate: 0.5-1.0 Gy/s) irradiation, followed by tissue collection at 96 hours post-irradiation for histological analysis. (B) Representative confocal images of jejunal sections from non-irradiated, Proton CONV, and Proton FLASH-irradiated mice showing Lgr5+ intestinal stem cells (green) and tdTomato+ (red). In-sets highlight higher magnification views of Lgr5+ crypts. (C) Violin plot demonstrates quantification of Lgr5+ crypts per field shows significantly higher stem cell preservation in Proton FLASH-irradiated mice compared to Proton CONV-irradiation. Data presented as mean ± SD. (Significant; **p < 0.005, ***p < 0.0005). n= 5 mice per group.*

 ***Supplementary Figure 9:*** *Proton FLASH irradiation preserves proliferative cell populations intestinal epithelium. (A) Light microscopic images of jejunal sections subjected to Ki67 immunostaining at 96 hours post-irradiation. B. Violin plot showed quantification of number of Ki67+ proliferating cells per intestinal crypt (50 well-defined crypts were quantified per mouse, in a minimum of 10 fields per slide). Proton FLASH significantly preserved Ki67⁺ proliferating cells compared to Proton CONV irradiation. Data are presented as mean ± SD, (Significant; ****p < 0.00005). n= 5 mice per group.*

 ***Supplementary Figure 10:*** *Single-cell transcriptomic analysis reveals different intestinal epithelial cell populations clusters. (A) UMAP plot shows Epithelial cells were identified by Epcam expression. (B)UMAP plot showing representation of 24 epithelial cell clusters identified from intestinal cells. (C) representation of Dot plot of selected marker genes across epithelial clusters, with dot size representing percentage of expressing cells and color indicating average expression. (D) Representation of CREPT/RPRD1B expression across all the epithelial cells clusters. (E) representing CBCs have highest Stemness score overall in all the epithelial cells clusters.*

 ***Supplementary Figure 11:*** *Proton FLASH reduces oxidative stress in intestinal epithelium. (A) Confocal microscopic images of mouse jejunal sections to detect epithelial cells expressing OXPHOS proteins at 24 hours post-irradiation. Cells expressing OXPHOS protein (stained purple in merged image) were indicated with arrow. Nuclei are stained (blue) with Dapi. (B) Violin plot demonstrated increase in OXPHOS signal in Proton CONV irradiation compared to non-irradiated controls (No IR), whereas Proton FLASH irradiation resulted in a reduced OXPHOS intensity compared to Proton CONV irradiation. OXPHOS positive signal was quantified by Image J and expressed as Mean Fluorescent Intensity (%). Data are expressed as mean ± SD. (Significant; **p < 0.005, ***p< 0.00005). n= 5 mice per group.*

***Supplementary Figure 12:*** *Proton FLASH reduces radiation induced ROS production in intestinal tissue. (A) Confocal microscopic images of mouse jejunal sections stained with Dihydroethidium (DHE) to detect tissue ROS level at 24 hours post-irradiation. Cells producing ROS appeared as purple (indicated with arrow) by producing 2-hydroxyethidium (emitting red fluorescence). Nuclei are stained (blue) with Dapi. (B) Violin plot demonstrated increase in fluorescence signal representing ROS in mice exposed to Proton CONV irradiation compared to non-irradiated controls (No IR), whereas Proton FLASH irradiation resulted in a reduced ROS signal compared to Proton CONV irradiation. ROS positive signal was quantified by Image J and expressed as Mean Fluorescent Intensity (%). Data are presented as mean ± SD. (Significant; **p < 0.005, ***p < 0.0005). n= 5 mice per group.*

***Supplementary Figure 13:*** *Proton FLASH reduces DNA damage assessed by γ-H2AX foci formation.* *(A) Light microscopic images of jejunal sections subjected to γ-H2AX immunostaining at 24 hours post-irradiation. (B) Violin plot demonstrated quantification of γ-H2AX foci+ cells per crypt, representing DNA double-strand break formation. Proton CONV irradiation with 21 Gy (dose rate: 0.5-1.0 Gy/s) induced a robust increase in γ-H2AX foci compared to non-irradiated controls (No IR), while Proton FLASH irradiation (dose rate: 57.14-64.29 Gy/sec.) significantly attenuated DNA damage relative to Proton CONV irradiation. Arrow indicated γ-H2AX foci+ cells in a crypt. (a total of 30 well-defined crypts were quantified per mouse) .Data are shown as mean ± SD. (Significant; ****p < 0.00005). n= 5 mice per group.*

***Supplementary Figure 14:*** *Fecal microbiota transfer from Proton FLASH exposed mice mitigated radiation induced intestinal injury in recipient mice exposed to Proton CONV abdominal irradiation. (A) Schematic representation of experimental set-up showing fecal microbiota transplant (FMT) from donor mice into recipient mice followed by tissue collection for histological analysis at day 4 post-irradiation. (B) Representative H&E-stained jejunal sections from* *Proton CONV-irradiated recipients (18 Gy with dose rate: 0.5-1.0 Gy/sec.) receiving No IR FMT, Proton CONV FMT (18 Gy with dose rate: 0.5-1.0 Gy/sec.), or Proton FLASH FMT (18 Gy with dose rate: 57.14-64.29 Gy/sec.), show improved mucosal preservation in Proton FLASH FMT group compared with No IR or Proton CONV FMT group. (C-D) Violin plot demonstrating quantification of villus length and crypt depth. Proton FLASH-FMT receiving mice tissue demonstrated significantly higher villi length and crypt depth compared to No IR or Proton CONV FMT groups, while no significant difference was observed between No IR and Proton CONV FMT groups. (E) showing the number of Ki67+ proliferative cells per crypt. (F) Violin plot demonstrates quantification of Ki67+ cells per crypt. Proton FLASH-FMT receiving mice demonstrate significantly higher Ki67+ cell counts per crypt compared to Proton CONV-FMT or No IR FMT group . Data are shown as mean ± SD. (Significant; **p < 0.005; ***p < 0.0005; ****p < 0.00005, NS: not significant). n=5 mice per group.*

***Supplementary Figure 15:*** *Mice abdominal irradiation exposure setup. Representative images showing the experimental setup for localized abdominal irradiation using Proton FLASH/ Proton CONV irradiation to mice. The mouse is placed in a custom-designed jig and only the abdominal region is exposed to radiation. Crosshair laser alignment is used for precise targeting of the abdominal field. Multiple views demonstrate the positioning of the mouse, and beam field alignment.*

***Supplementary Figure 16:*** *Assessment of cage effects on gut microbial population composition in non-irradiated (No IR) control mice. Principal coordinate analysis (PCoA) based on Bray Curtis dissimilarity of relative microbial abundance was performed on fecal samples from non-irradiated control mice, with samples colored by housing cage. The lack of distinct clustering by cage indicates minimal cage-associated variation in microbial populations. PERMANOVA analysis confirmed, No significant cage effect (p = 0.393). n=10 mice per group.*

***Supplementary Tabel 1:***
